# Single-Patient Molecular Testing with NanoString nCounter Data Using a Reference-Based Strategy for Batch Effect Correction

**DOI:** 10.1371/journal.pone.0153844

**Published:** 2016-04-20

**Authors:** Aline Talhouk, Stefan Kommoss, Robertson Mackenzie, Martin Cheung, Samuel Leung, Derek S. Chiu, Steve E. Kalloger, David G. Huntsman, Stephanie Chen, Maria Intermaggio, Jacek Gronwald, Fong C. Chan, Susan J. Ramus, Christian Steidl, David W. Scott, Michael S. Anglesio

**Affiliations:** 1 Department of Pathology and Laboratory Medicine, University of British Columbia, Vancouver, Canada; 2 Department of Women’s Health, University Hospital Tuebingen, Tuebingen, Germany; 3 British Columbia Centre for Disease Control, Vancouver, Canada; 4 Genetic Pathology Evaluation Centre (GPEC), Vancouver General Hospital and The University of British Columbia, Vancouver, Canada; 5 Department of Statistics, University of British Columbia, Vancouver, Canada; 6 Pancreas Centre BC, Vancouver, Canada; 7 Department of Obstetrics and Gynaecology, University of British Columbia, Vancouver, Canada; 8 Department of Preventive Medicine, Keck School of Medicine, University of Southern California, Los Angeles, United States of America; 9 School of Women's and Children's Health, University of New South Wales, Sydney, NSW, Australia; 10 Department of Genetics and Pathology, International Hereditary Cancer Center, Pomeranian Medical University, Szczecin, Poland; 11 Centre for Lymphoid Cancer, British Columbia Cancer Agency Cancer Research Centre, Vancouver, Canada; 12 The Kinghorn Cancer Centre, Garvan Institute of Medical Research, Darlinghurst, NSW, Australia; Princess Margaret Cancer Centre, CANADA

## Abstract

A major weakness in many high-throughput genomic studies is the lack of consideration of a clinical environment where one patient at a time must be evaluated. We examined generalizable and platform-specific sources of variation from NanoString gene expression data on both ovarian cancer and Hodgkin lymphoma patients. A reference-based strategy, applicable to single-patient molecular testing is proposed for batch effect correction. The proposed protocol improved performance in an established Hodgkin lymphoma classifier, reducing batch-to-batch misclassification while retaining accuracy and precision. We suggest this strategy may facilitate development of NanoString and similar molecular assays by accelerating prospective validation and clinical uptake of relevant diagnostics.

## Introduction

The use of molecular technologies in clinical assays for guiding patient diagnosis, prognosis and management is paving the way for precision medicine. Gene expression in particular has been widely used in biomedical research to identify biomarkers and genetic profiles that enable disease diagnosis, (sub) classification, and prediction of prognosis and response to therapy[1–4]. Despite advances in molecular research, clinical adoption of gene expression assays has been slow. This is proposed to be, in part, due to the impact of technological and biological biases that arise during sample collection and processing, resulting in poor reproducibility[5–7].

Pre-processing, normalization and accounting for systematic sources of variability in gene expression data affect the ability to combine different cohorts for model development and cross-cohort predictions for a single patient; these are key requirements for the development, validation and proof of utility for clinical assays[8,9]. Batch effects (BE) refer to the systematic and technical variations between measurements introduced when handling samples in batches. BE are ubiquitous in gene expression analysis[8], and their presence could mask or simulate biological signals in data, resulting in either spurious and/or missed associations, especially when the biological factor of interest is confounded with a given batch[10].

Approaches for BE adjustment in multi-sample data [11–15] generally assume homogeneity amongst populations across batches, which is often not the case in practice. In addition, multi-sample methods are impractical in clinical settings, where patient samples are typically collected in small numbers, often one at a time, making single-patient data processing vital to the translation of molecular assays. Several studies have suggested the use of a reference-based approach [9,11] for BE adjustments, resulting in quantification relative to reference sample(s) that are run alongside clinical specimens. Depending on the end goal, types of reference samples may include RNA pooled from actual samples or cell lines, DNA oligonucleotides, synthetic RNA, or universal human RNA[16].

Generally, BE refers to systematic variability that may be attributed to RNA extractions, types or conditions of tissue, differences between operating labs and technicians, the use of different batches of reagents, age and storage conditions of assays components, experimental conditions, or hardware versions. Failing to correct for BE, particularly when the signal from the confounder is larger than the biological signal, can result in missed or false discoveries and irreproducible results[9,17,18]. Good experimental design can alleviate the impact of such confounding, subject to logistics and cost constraints. Additionally, if the sources of BE are not obvious, they may go unmeasured and unaccounted for.

The NanoString nCounter technology is a relatively new platform for quantifying RNA that exhibits several advantages over traditional microarray, quantitative PCR (qPCR) and RNA-seq methods. It is simple to use, highly automated, cost- and time-effective allowing quantification of up to 800 targets in a single reaction. Its sensitivity is comparable to that of qPCR[19–22], with the advantage that RNA content is measured directly, without amplification or other enzymatic processing. NanoString has been shown to work well even when nucleic acids are degraded[22,23], as is usually the case with formalin-fixed paraffin-embedded (FFPE) tissues, the mainstay of pathology labs worldwide. This feature facilitates large retrospective clinical studies, making NanoString a popular platform for the development and validation of prognostic and diagnostic assays[24–26]. Validated assays would have the potential for near immediate transfer to clinical use without the need to modify standard pathology handling.

Using the NanoString nCounter platform, we considered quality control metrics, normalization procedures, and BE adjustment methods for gene expression data obtained with the goal of assembling a protocol suitable for single-patient processing, which can be adopted in clinical settings from start to finish. We assessed both within and between-batch variability, focusing on the latter as many existing studies have already investigated within-batch reproducibility of nCounter data[20,23,27]. Different types of references were considered for BE adjustment and compared to multi-sample approaches. The impact of BE correction with a reference-based method was illustrated on the downstream analysis of a pre-existing Hodgkin lymphoma prognostic model[26].

## Materials and Methods

### NanoString nCounter Data

The NanoString technology is based on single-molecule imaging of color-coded barcodes bound to target-specific probes[8,10]. A CodeSet is a unique, single production-run, multi-assay mix of all probes of interest; it includes six positive controls, spiked-in at fixed, proportional concentrations (from 0.125–128 fM), and eight negative controls (probes without a corresponding target) used to assess background and non-specific binding. “Housekeeping genes” (HK) are an obligate component in the design of each CodeSet. Similar to quantitative RT-PCR, HK are expected to remain constant between biological conditions of interest and are used to control for the amount of RNA in a given reaction. A standard cartridge holds 12 lanes, allowing for 12 samples to be processed in a given run.

### Cohort Description

Datasets obtained from different NanoString experiments included 161 clinical specimens from two cancer types: Hodgkin lymphoma (HL) and ovarian cancer (OC). For the HL cohort, data was derived from Scott *et al*.[26], wherein a subset of samples had replicate data generated on a second CodeSet (n = 32) and an additional subset had data generated on a third CodeSet (n = 10). OC specimens (n = 129) were obtained through the OVCARE tissue bank and the Ovarian Tumour Tissue Analysis (OTTA) consortium and were also run on two CodeSets. Ovarian cancer cell lines (OVCL)[28] were also run in duplicate across CodeSets (n = 13). The last two cohorts were DNA oligonucleotides, complementary to target RNA in the HL (HLO) and the OC (OVO) CodeSets, run at different concentrations for a total of 203 runs. A detailed description of individual cohorts is given in Table 1. All specimens were collected through hospital based research studies with approval of local research ethics and/or institutional review boards. The BCCA/UBC research ethics board further approved generation of gene expression data for investigation of tumour biology, including development of RNA-based classifiers.

**Table 1:** Cohort Description.

*Hodgkin Lymphoma Clinical Samples (HL)*		
**CodeSet**	**# Runs**	**Type**
HL1	32	Unique Samples
HL2	32	Replicates of HL1 samples
HL3	10	Replicates (subset of HL1 samples)
*TOTAL*	*74*	
*Ovarian Cancer Clinical Samples (OC)*		
**CodeSet**	**# Runs**	**Type**
OC1	129	Unique
OC2	129	Replicates of OC1 samples
*TOTAL*	*258*	
*Ovarian Cancer Cell Lines (OVCL)*		
**CodeSet**	**# Runs**	**Type**
OC1	13	Unique
OC2	13	Replicates of OC1 samples
*TOTAL*	*26*	
*DNA Oligonucleotides for the HL CodeSet (HLO)*		
**CodeSet**	**# Runs**	**Type**
HL1	36	HLO pool run at different concentrations
HL2	30	HLO pool run at different concentrations
HL3	2	HLO pool run at different concentrations
*TOTAL*	*68*	
*DNA Oligonucleotides for the OC CodeSet (OVO)*		
**CodeSet**	**# Runs**	**Type**
OC1	47	OVO pool run at different concentrations
OC2	88	OVO pool run at different concentrations
*TOTAL*	*135*	

### Nucleic acid preparation

RNA extraction was standardized within each research group (either Hodgkin or ovarian). All samples were reviewed for the presence of tumour. Ovarian frozen specimens were cryosectioned (5–40 section at 20 μm, depending on face size), tissue was dissociated by vortexing in Trizol reagent and then processed using the Qiagen miRNeasy protocol (Qiagen) as per manufacturer’s recommendations. FFPE tissues were sectioned (3 sections at 10 μm) into microfuge tubes. OTTA-sourced specimens were collected by scrapping 2–4 5um section off glass slides. FFPE specimens were de-paraffinized and processed according to the Qiagen miRNeasy FFPE kit (Qiagen) with an elongated 55°C digest period (45 min). Lymphoma specimens, all FFPE derived, were processed similarly with the exception of deparaffinization using Qiagen deparaffinization solution as described previously[26].

For oligonucleotide experiments, standard desalted, single strand, 100-base DNA oligonucleotides were obtained (IDT) and a single pool of DNA oligonucleotides was generated by mixing all oligonucleotides in equimolar concentration corresponding to the sum of targets in both ovarian CodeSets. This resulted in each CodeSet targeting only a subset of the total pool. We refer to the “functional concentration” as the concentration of the subset oligonucleotide pool that is targeted in a given CodeSet hybridization. In the case of Hodgkin lymphoma CodeSets an oligonucleotide pool corresponding only to the prognostic genes [26] was used.

### NanoString RNA expression data

The manufacturer’s protocol was adhered to with the exceptions noted below. Briefly, capture, reporter, specimen total RNA (or DNA oligonucleotides) were mixed with hybridization buffer and hybridized at 65°C overnight. Sample, wash reagents and imaging cartridge were then processed on the nCounter Prep Station and finally imaged on the nCounter Digital Analyzer. FFPE derived specimens used 200–500 ng of input total RNA, whereas frozen specimens used only the recommended 100 ng. Hybridization times ranged from 12–23 hours and were strictly monitored in selected cohort experiments. See Table 1 for further details.

### Statistical Methods

Raw data was assessed using several quality assurance (QA) metrics to measure imaging quality, oversaturation and overall signal to noise. All samples satisfying QA metric checks were log-transformed (base 2) to help with distributional assumptions, and were normalized by subtracting the average expression level of HK genes (equivalent to the geometric mean normalization on the raw scale). Principal Components Analysis (PCA) and Principal Variance Components Analysis (PVCA) were used to measure the impact and assess sources of variability in the normalized data. The minimal set of principal components for PVCA[29] were selected to ensure at least 60% explained variability was retained. We compared the intra-CodeSet to inter-CodeSet variability using the Dispersion Separability Criterion (DSC)[30], a ratio of between- to within-batch dispersion computed from batch scatter matrices. High DSC (over 0.5) indicates large inter-batch variability. Significance of DSC was assessed using a permutation test: starting with a null hypothesis of no batch effects present and that the data is homogeneous in terms of batches—five thousand permutations were generated from the full data (including all batches). DSC values were computed for each permutation; at the end of all the runs the proportion of values greater than the observed DSC is computed to yield the p-value (significance level at 0.05).

A series of multi-sample methods were applied to both OC and HL data: batch mean centering (bmc)[11], mean and variance standardization (zscore)[11], a two-stage location/scale correction (ber)[11], and parametric empirical Bayes approach (combat)[14]. In addition, we considered different types of references and their performance in comparison to multi-sample methods. HL samples were calibrated using the HLO data (HLO_ref) and three clinical HL samples chosen at random (any3_ref). Similarly, OC samples were adjusted using the OVO data (OVO_ref), five samples from each carcinoma histotype[31] chosen at random (any5_ref), and the OVCL data (OVCL_ref; averaging over all OC cell lines). PVCA plots were used to compare the variability post-adjustment to the unadjusted data. Furthermore, gene-wise agreement between CodeSets was compared using: Pearson’s correlation coefficient (*R*), for precision; the coefficient of accuracy (C_a_), to assess the systematic bias; Lin’s concordance correlation[32] was used to capture both precision and accuracy simultaneously (R_c_ = *R* C_a_). The degree of BE removal was evaluated using DSC.

Adjusting for batch effects using reference samples, involved running designated reference samples in every batch along actual samples and measuring the same set of genes. Assuming we have two batches (A and B) and were interested in calibrating samples *X*^*B*^ that were run in batch B to samples in *X*^*A*^ that were run in batch A, the reference approach would require that some number of reference samples (R) would be run in both batches A and B, resulting in R^*A*^ and R^*B*^. These reference samples would be used to calibrate between the two batches. Mathematically, we assumed the following standard additive (on the log scale) batch effect model on the normalized and log (base 2) transformed NanoString data[11]:

$$X_{ij}^{A}=X_{ij}^{\prime }+b_{ij}^{A}+\varepsilon_{ij}^{A}$$ (1)

Where ${\text{X}}_{ij}^{A}$ is the observed mRNA expression of gene *i* in sample *j* run in batch A, ${\text{X}}_{\text{ij}}^{\text{'}}$ is the true gene expression, ${\text{b}}_{\text{ij}}^{\text{A}}$ is the batch effect corresponding to batch A, and $\epsilon_{\text{ij}}^{\text{A}}$ is a random error assumed to be independent with expected value equal to 0. Let R^*A*^ be a set of designated reference samples of size k, run alongside actual samples in batch A, we can then write

$$R_{il}^{A}=R_{il}^{\prime }+b_{il}^{A}+\varepsilon_{il}^{A},$$ (2)

and to remove BE, we subtract the observed gene expression of the reference from the observed gene expression of clinical samples

$${\hat{X}}_{ij}=\;X_{ij}^{A}-\frac{1}{k}\sum_{l=1}^{k}R_{il}^{A}$$ (3)

We note in the above equation that if a certain gene *i*, measured in the original sample, is not expressed in the reference sample, then $\sum_{l=1}^{k}{\text{R}}_{\text{il}}=0$, thus resulting in no adjustment for that gene and BE will not be eliminated. For this reason, it is important to ensure that the selected reference samples have a good expression level of the genes of interest otherwise the reference will not be effective at eliminating BE.

If the interest is to combine data obtained from different batches, this can be achieved by subtracting RA=∑l=1krilAk from X^A^ and similarly RB=∑l=1krilBk from X^B^ prior to combining the two data sets. In certain cases a model may have been developed with data that were not batch corrected and hence future data would need to be calibrated to the training data. This is achieved by adding the difference between the reference to the new data: *X*^*B*^ + (*R*^*A*^ − *R*^*B*^).

## Results

### Quality Assurance Metrics

**Fig 1:**
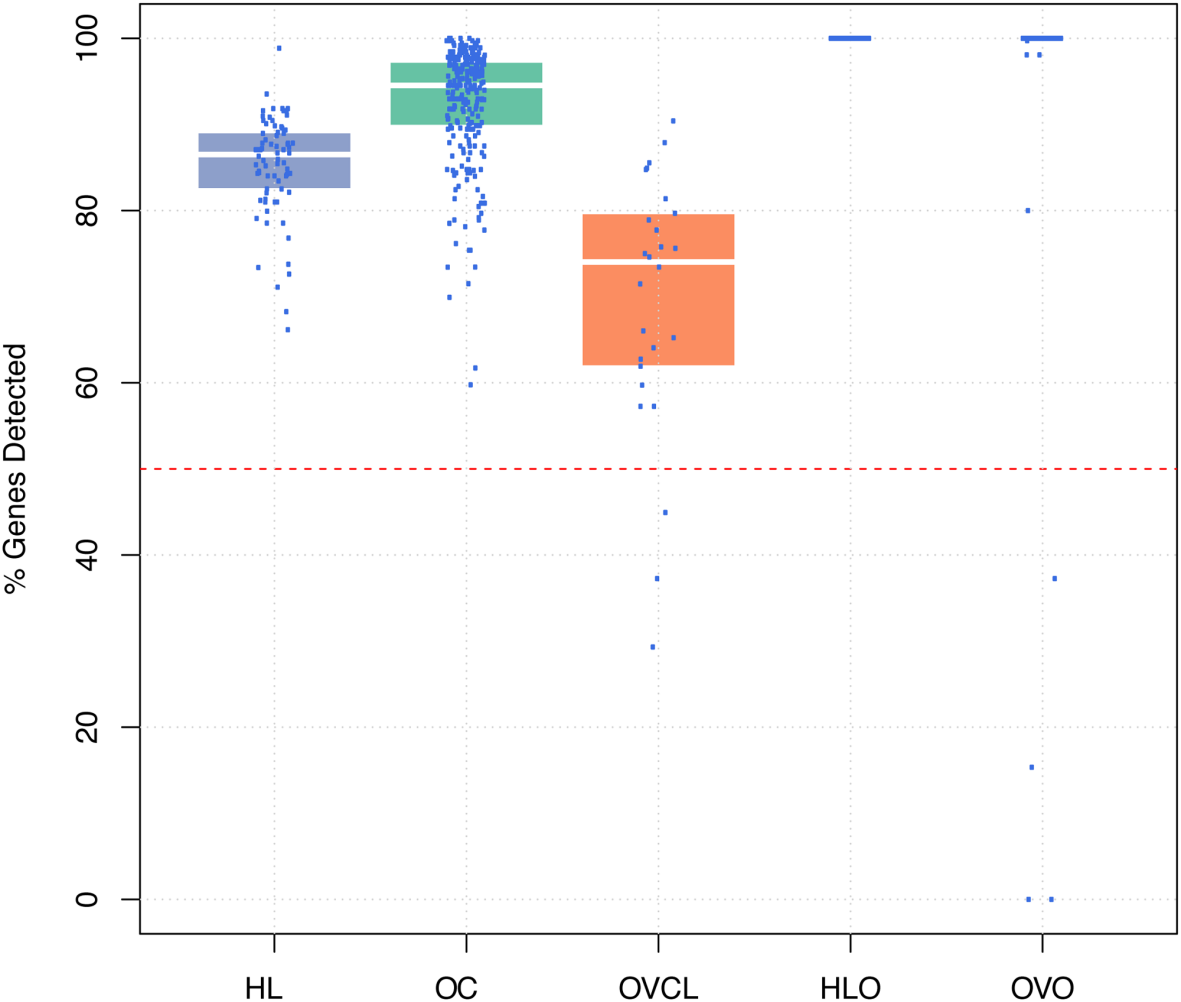
Percentage of genes detected above the limit of detection (LOD) by cohort. Each point on the boxplot represents a NanoString nCounter unique run (duplicates and triplicates included where available). The colored boxes represent the distribution of the percentage of genes detected in a particular cohort. The white line indicates the median. A cutoff of 50% was used for Cell Lines and clinical samples, and 95% was used for oligonucleotide samples. HL: Hodgkin lymphoma clinical samples, OC: ovarian cancer clinical samples, OVCL: ovarian cancer cell lines, HLO: oligonucleotides corresponding to the HL CodeSet, OVO: oligonucleotides corresponding to the OC CodeSet.

**Fig 2:**
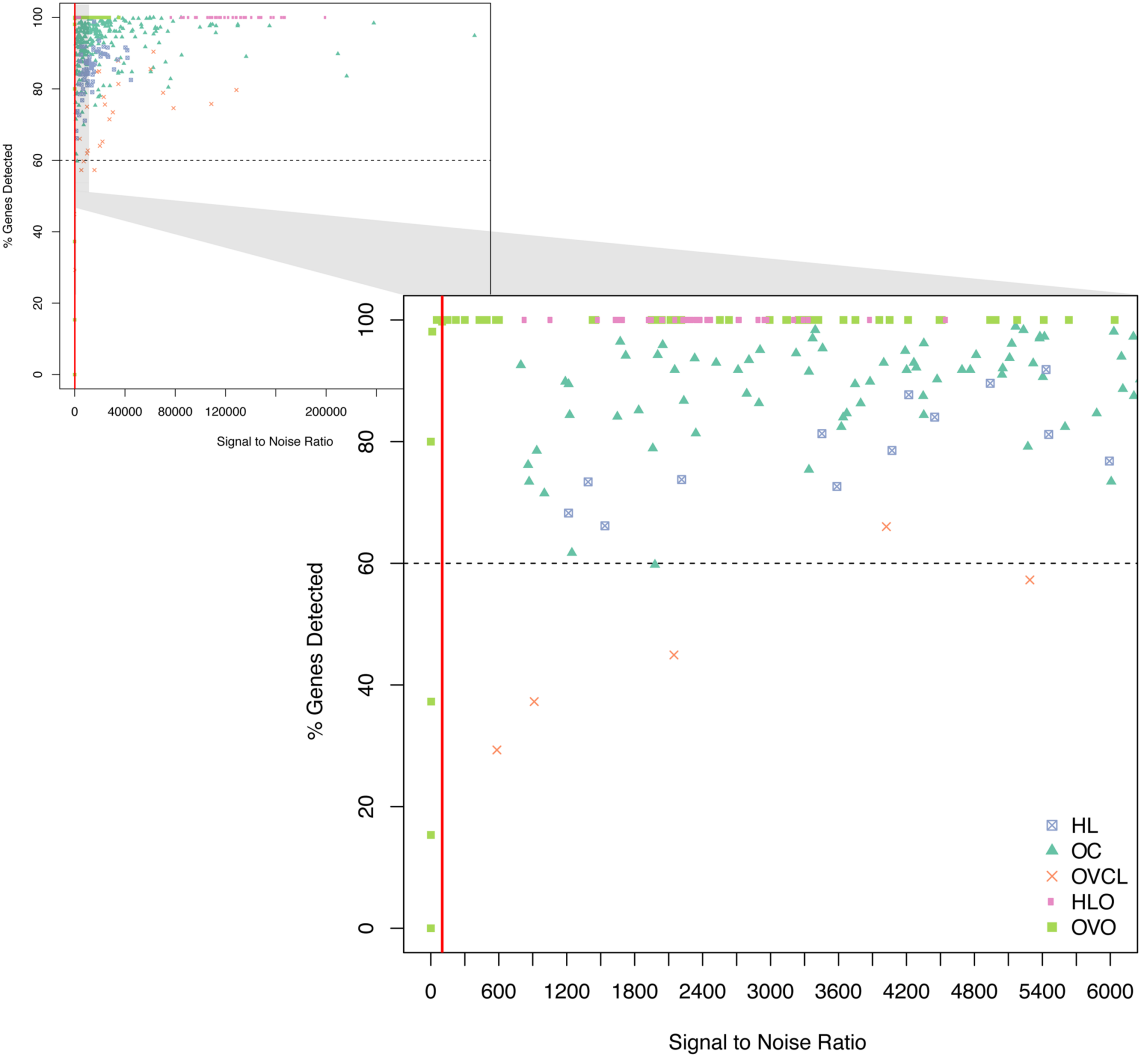
Percentage of genes detected as a function of Signal to Noise Ratio by cohort. Each point on the plot represents a NanoString nCounter unique run (duplicates and triplicates included where available). The zoomed in section illustrates how the selected cut-off excludes samples that have low signal to noise and low % genes detected. HL: Hodgkin lymphoma clinical samples, OC: ovarian cancer clinical samples, OVCL: ovarian cancer cell lines, HLO: oligonucleotides corresponding to the HL CodeSet, OVO: oligonucleotides corresponding to the OC CodeSet.

All OC clinical samples passed all QA metrics. Only one HL sample failed based on fields of view (FOV; obtained 73% cutoff criteria >75%) metric and was removed. Samples were flagged as imaging failures if the percentage of lane images FOV obtained was less than 75% of the requested number of fields. This may be affected by either physical problems with the cartridge or oversaturation of probes (locally or across the whole lane). Oversaturation occurs when probes compete for physical space on a lane, displacing positive control probes and resulting in loss of linearity while simultaneously probes expressed at low levels become indistinguishable from background noise. Lane oversaturation was determined using the linearity of positive control probes (R^2^< 0.95) and the ability to detect the smallest positive control. Failures were more common in OVO cohort (12/135 failed) vs. HLO (no failure); however, this was expected as dilution experiments were set to stress usability limits. OVCL had a large number of genes that were not expressed (Fig 1), likely due to OC CodeSet design based on OC histotype and molecular subtype analysis[31,33,34], with a substantial proportion of stromally-derived genes[33,34]. Taking this into account, we were less stringent in excluding cell lines that had a lower percent of genes detected above background. Clinical samples with a signal-to-noise ratio (S/N) smaller than 100 and <50% genes detected above limit of detection (LOD) were considered poor quality and removed from analyses. Oligonucleotide samples with less than 95% detection levels were considered failed as these synthetic samples were engineered for 100% perfect-match. The S/N, computed as a ratio of the geometric mean of HK and the LOD, was used as an overall sample quality measure. LOD is a metric used to determine the level of background noise in the system; high LOD results in fewer genes being detected above background noise. The fraction of genes above LOD is a good measure of the quality of RNA preparation and the quantity of RNA loaded, however it should be noted this is not a generalizable cut-off and must be established (empirically) for a given codeset and experimental design. A subset of OVO cohort also failed the S/N metric; again, this was expected as noted above (Fig 2). Samples with high S/N and a large % of genes detected are generally considered better quality samples; however, the cutoff for the removal of samples from analyses would be application-dependent. The results are presented in Table 2 and more details are provided in S1 File.

**Table 2:** Overall QC Measures by cohort.

	Total	HL	OC	OVCL	HLO	OVO
**Total**	561(100%)	74 (13%)	258 (46%)	26 (5%)	68 (12%)	135 (24%)
**All QC**						
Failed	15 (3%)	1 (1%)	0 (0%)	2 (8%)	0 (0%)	12 (9%)
Passed	546 (97%)	73 (99%)	258 (100%)	24 (92%)	68 (100%)	123 (91%)
**FOV**						
Failed	6 (1%)	1 (1%)	0 (0%)	0 (0%)	0 (0%)	5 (4%)
Passed	555 (99%)	73 (99%)	258 (100%)	26 (100%)	68 (100%)	130 (96%)
**Linearity**						
Failed	2 (0%)	0 (0%)	0 (0%)	0 (0%)	0 (0%)	2 (1%)
Passed	559(100%)	74 (100%)	258 (100%)	26 (100%)	68 (100%)	133 (99%)
**Smallest PC**						
Failed	2 (0%)	0 (0%)	0 (0%)	0 (0%)	0 (0%)	2 (1%)
Passed	559(100%)	74 (100%)	258 (100%)	26 (100%)	68 (100%)	133 (99%)
**S/N**						
Failed	12 (2%)	0 (0%)	0 (0%)	2 (8%)	0 (0%)	10 (7%)
Passed	549(98%)	74 (100%)	258 (100%)	24 (92%)	68 (100%)	125 (93%)

All % are column percentages. *Columns*: HL: all Hodgkin Lymphoma clinical samples; OC: all ovarian cancer clinical samples; OVCL: ovarian cancer cell lines; HLO: DNA oligonucleotides corresponding to the HL CodeSet; OVO: DNA oligonucleotides corresponding to OC CodeSet. *Rows*: All QC: all quality control metrics; FOV: fields of view metric; Linearity: Linearity of positive control metric; Smallest PC: detection of smallest positive control metric; S/N: signal to noise ratio metric.

Binding density (BD), a quality assurance measure suggested by the manufacturer to monitor oversaturation, was not used, as there were minor inconsistencies between hardware versions. BD also did not correlate well with S/N (See S1 File for detail), suggesting BDs well above the recommended limit were still valid for quantitation.

### Data Normalization

NanoString generates non-amplified count data, which may be right-skewed depending on gene selection. All samples satisfying QA metric checks were log-transformed (base 2) to help with distribution assumptions, as needed. NanoString recommends a three-step normalization procedure which involves adjustments relative to positive controls and HK as well as background subtraction [35]. Different normalizations for nCounter data have also been considered previously[20]. We found that normalizing to positive controls made little difference and resulted in unnecessary processing of the data. Similarly, estimating and subtracting background resulted in a “false floor”, a problem that becomes most evident when comparing data from different CodeSets. Our analysis demonstrated that a sufficient step is the subtraction of the arithmetic mean of HK (on the log scale, this is equivalent to the geometric mean on the raw scale; S2 File). Finally, for the methodology to be suitable in a single-patient clinical setting, we strongly discourage rescaling, which creates an unnecessary dependency on the level of expression of other samples processed concurrently. Using our approach, the final data can be interpreted as a relative fold change to [the mean of] HK.

HK selection, in the HL CodeSets was kept consistent with published works, using *ACTB*, *CLTC* and *RPLP0*. For OC, HK were chosen from a panel of commonly accepted invariant genes represented on the NanoString Human Reference GX panel, based on their variability from a number of OC studies[33,34]. Five genes were selected from the upper (2), middle (2), and lower (1) expression level quantiles: *RPL19*, *ACTB*, *PGK1*, *SDHA*, and *POLR1B* from highest to lowest median expression respectively. The selection of HK is important; optimal genes should have lower variance across samples and an expression level that spans that of all other genes while remaining in the linear dynamic range of the assay. Moreover, although there is little formal analysis as to the optimal number of genes to use as housekeepers, more is better[36]; our current preference is to recommend a minimum of 5.

### Monitoring Sources of Variability

**Fig 3:**
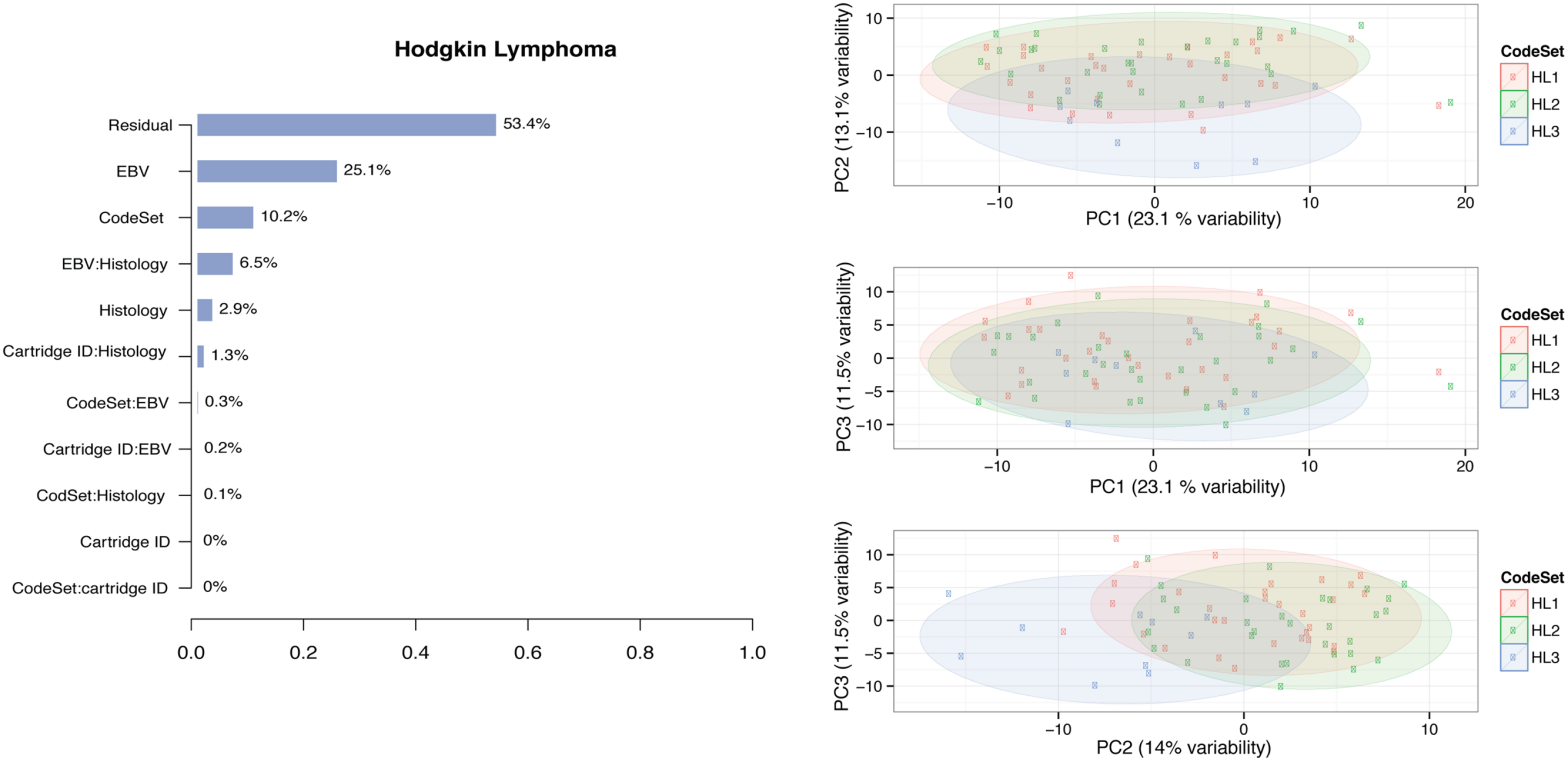
PVCA and PCA plots of the Hodgkin Lymphoma clinical samples. We considered the PVCA plot (A) of the HL clinical samples run in different batches. The percentages represent the variability explained by each factor and first order interaction between factors. The PCA plot (B) provides a two-dimensional summary of the pairwise plot of the first three principal components, which represent 49% of the variability in the data. HL1, HL2, and HL3 label each of unique CodeSets corresponding to the HL gene list.

**Fig 4:**
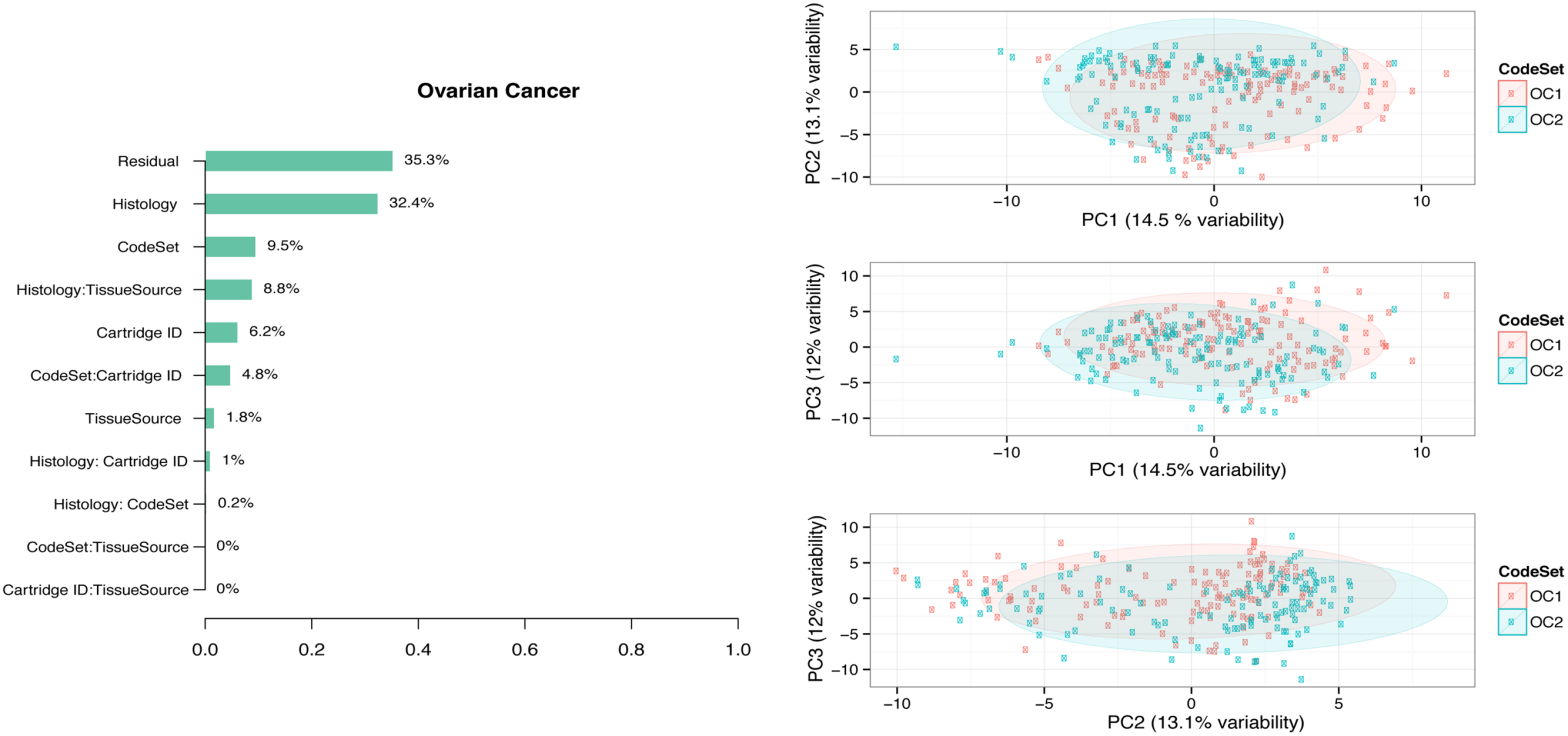
PVCA and PCA plots of the ovarian cancer clinical samples. We considered the PVCA plot (A) of the OC clinical samples run in different batches. The percentages represent the variability explained by each factor and first order interaction between factors. The PCA plot (B) provides a two-dimensional summary of the pairwise plot of the first three principal components, which represent 40% of the variability in the data. CS1, CS2, and CS3 label each of unique CodeSets corresponding to the OC gene list.

CodeSet variability can challenge the reproducibility of results for any third-party without access to original samples and CodeSet. Technical and true biological (e.g. histology and Epstein Barr Virus (EBV) status) variables were considered in PVCA (Figs 3 and 4).

In both OC and HL, the variability associated with CodeSet was ~10%. HL clinical samples showed pronounced CodeSet-dependent shifts in plots of the first three principal components. A significant DSC of 0.21 and 0.26 was found in the HL and OC datasets respectively (p-value <0.01) (Tables 3 and 4), indicating the presence of significant but weak BE. Finally, a gene-wise percent change in the average log-expression between CodeSets was computed. The median % change was around 10% in both the OC and HL gene sets, with certain genes being more stable across CodeSets than others (all compared genes had identical probe-sequence from lot-to-lot).

**Table 3:** Concordance between duplicates of HL clinical samples obtained from two CodeSets, after adjusting using different methods.

*Adjustment Method*	*R*_*c*_	*C*_*a*_	*R*	*DSC*	*p-value*
**No adjustment**	0.85	0.93	0.95	0.259	0.002
***Multi-Sample***					
**bmc**	0.94	1	0.95	0	1
**zscore**	0.95	1	0.95	0	1
**ber**	0.95	1	0.95	0.005	1
**pcombat**	0.92	0.99	0.95	0.073	0.96
***Single-Patient***					
**HLO_ref**	0.81	0.89	0.95	0.29	0
**any3_ref**	0.93	0.99	0.95	0.123	0.55

Column Labels: Rc: concordance coefficient; Ca: coefficient of accuracy; R: Coefficient of determination (Pearson’s correlation coefficient); DSC: Dispersion Separability Criterion.

**Table 4:** Concordance between duplicates of OC clinical samples obtained from two CodeSets, after adjusting using different methods.

*Adjustment Method*	*R*_*c*_	*C*_*a*_	*R*	*DSC*	*p-value*
**No adjustment**	0.88	0.97	0.92	0.21	0
***Multi-Sample***					
**bmc**	0.92	1	0.92	0	1
**zscore**	0.92	1	0.92	0	1
**ber**	0.92	1	0.92	0.002	1
**pcombat**	0.905	0.99	0.92	0.095	0.01
***Single-Patient***					
**OVCL_ref**	0.865	0.965	0.92	0.36	0
**OVO_ref**	0.875	0.97	0.92	0.191	0
**any3_ref**	0.91	1	0.92	0.076	0.13
**any5_ref**	0.91	1	0.92	0.057	0.64

Column Labels: Rc: concordance coefficient; Ca: coefficient of accuracy; R: Coefficient of determination (Pearson’s correlation coefficient); DSC: Dispersion Separability Criterion.

### Batch Effect Correction

Common multi-sample BE adjustments in gene expression experiments[11,37,13,12,15] typically require a generous sample set (n > 30) to be processed within each batch. Empirical Bayes methods[12,14] may be useful when the number of samples in each batch is small (n < 30); however, all these methods assume that the underlying biological feature representation is equal in every batch[12], a condition seldom met in clinical practice and observational studies.

Using reference samples for BE adjustment has been suggested as more effective method for calibration[9,11], where the expression level of the clinical sample is taken relative to the reference (see Materials and Methods).

**Fig 5:**
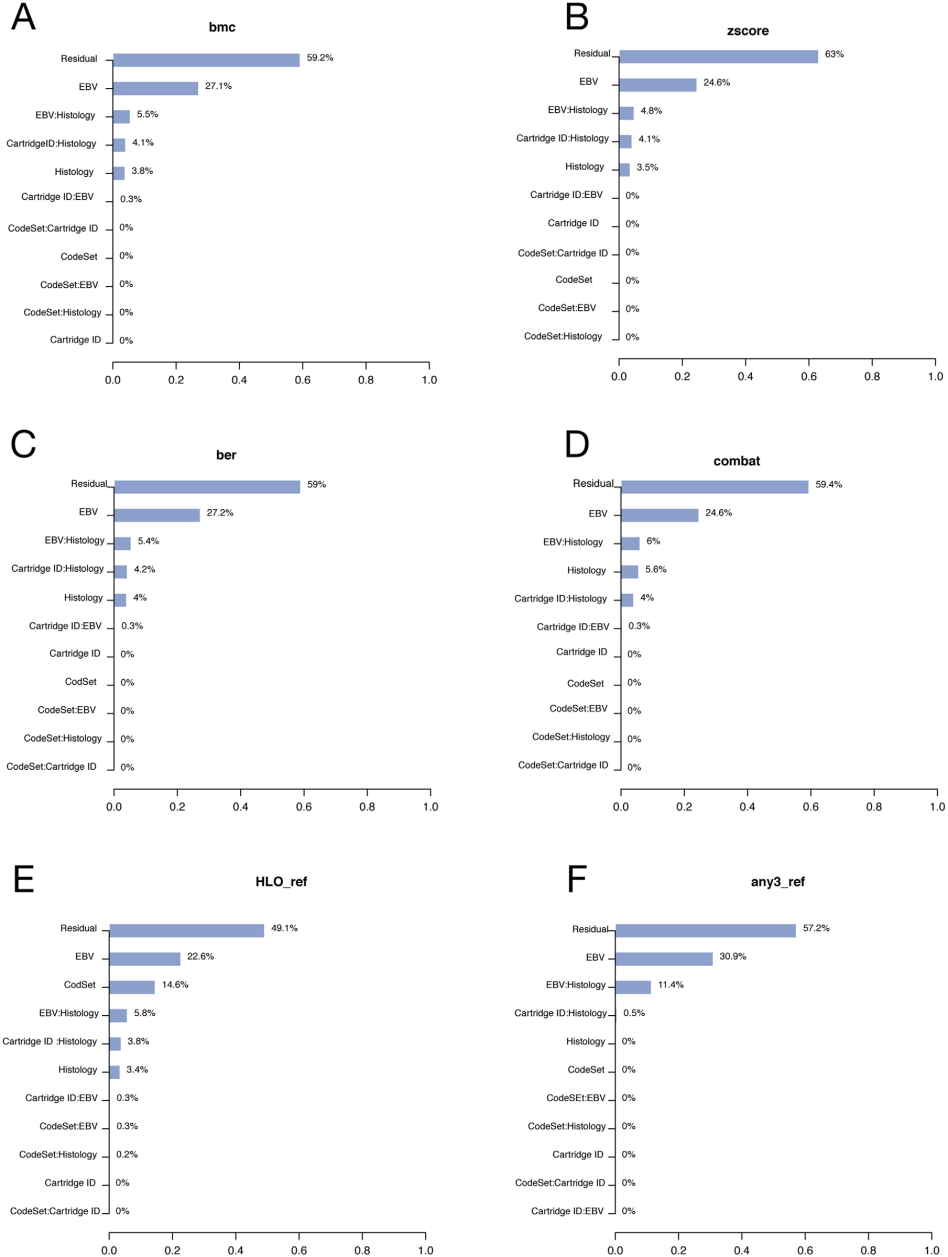
PVCA of the HL clinical samples after adjusting batch effect using different methods. We consider the PVCA plot of the HL clinical samples run in different batches after adjusting BE with different methods. In each plot, percentages represent the variability explained by each factor and first order interaction between factors.

**Fig 6:**
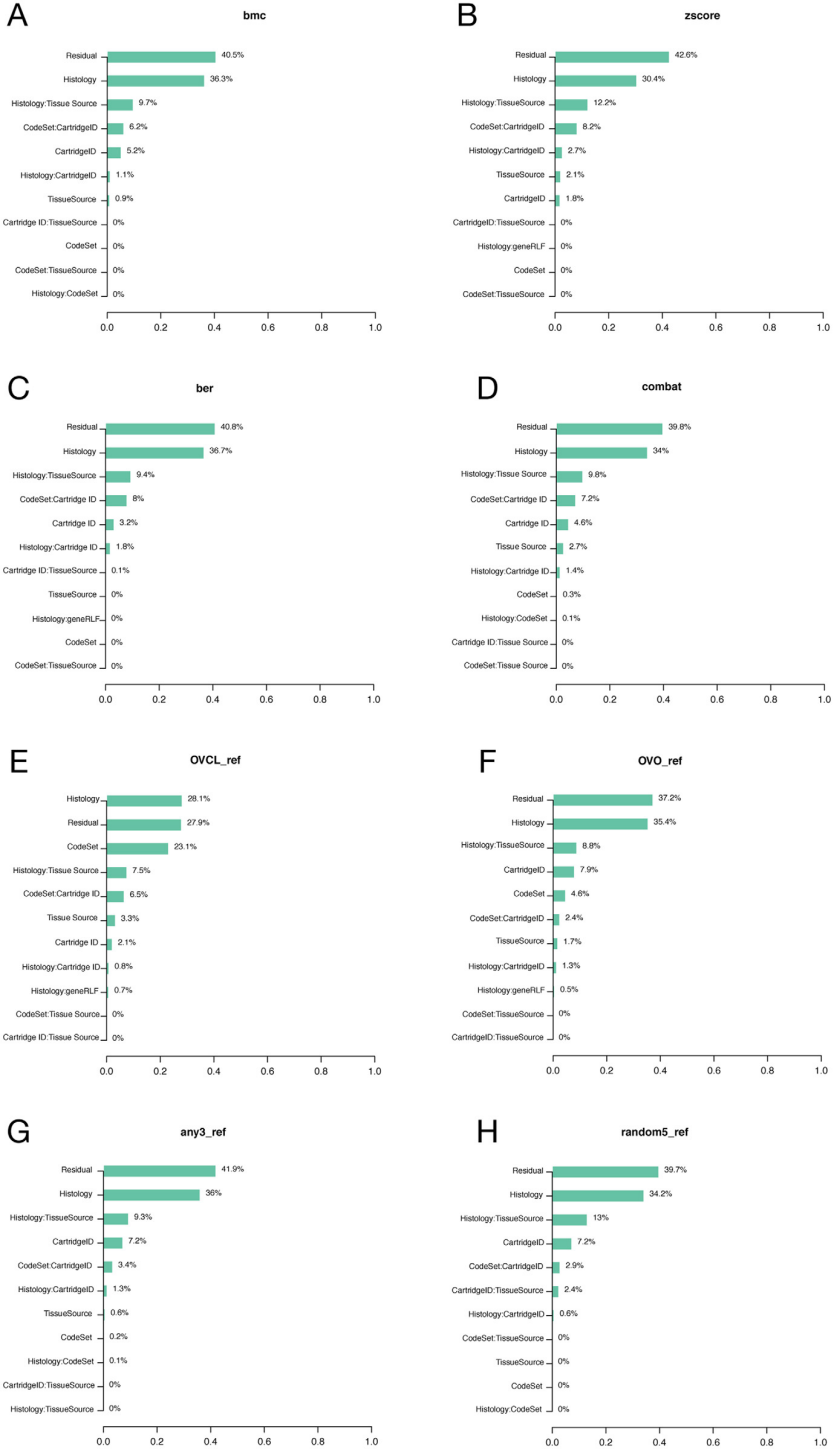
PVCA of the OC clinical samples after adjusting batch effect using different methods. We consider the PVCA plot of the OC clinical samples run in different batches after adjusting BE with different methods. In each plot, percentages represent the variability explained by each factor and first order interaction between factors.

PVCA plots were used to compare the variability post-adjustment to the unadjusted data (Figs 5 and 6). In the HL data, the batch effect due to CodeSet was removed in all cases except when using the HLO_ref. Similarly, in the OC data, % of variance due to CodeSet went down in all cases except in OVO_ref and OVCL_ref. The results (Tables 3 and 4) showed that for both cohorts, not correcting for BE, resulted in a smaller concordance correlation coefficient of 0.85 for the HL cohort and 0.88 for the OC cohort. Batch adjustment methods had no effect on precision; in contrast, accuracy was modified in all cases, with changes to the OC cohorts being marginal in comparison to HL. In multi-sample correction, all methods except combat in the OC cohort seemed to perform similarly in improving concordance between batches; DSC appeared to shrink in both cohorts after adjusting for BE. In reference-based methods, synthetic DNA oligonucleotides did not perform as expected, appearing to add more bias to the results.

### Downstream Analysis

**Fig 7:**
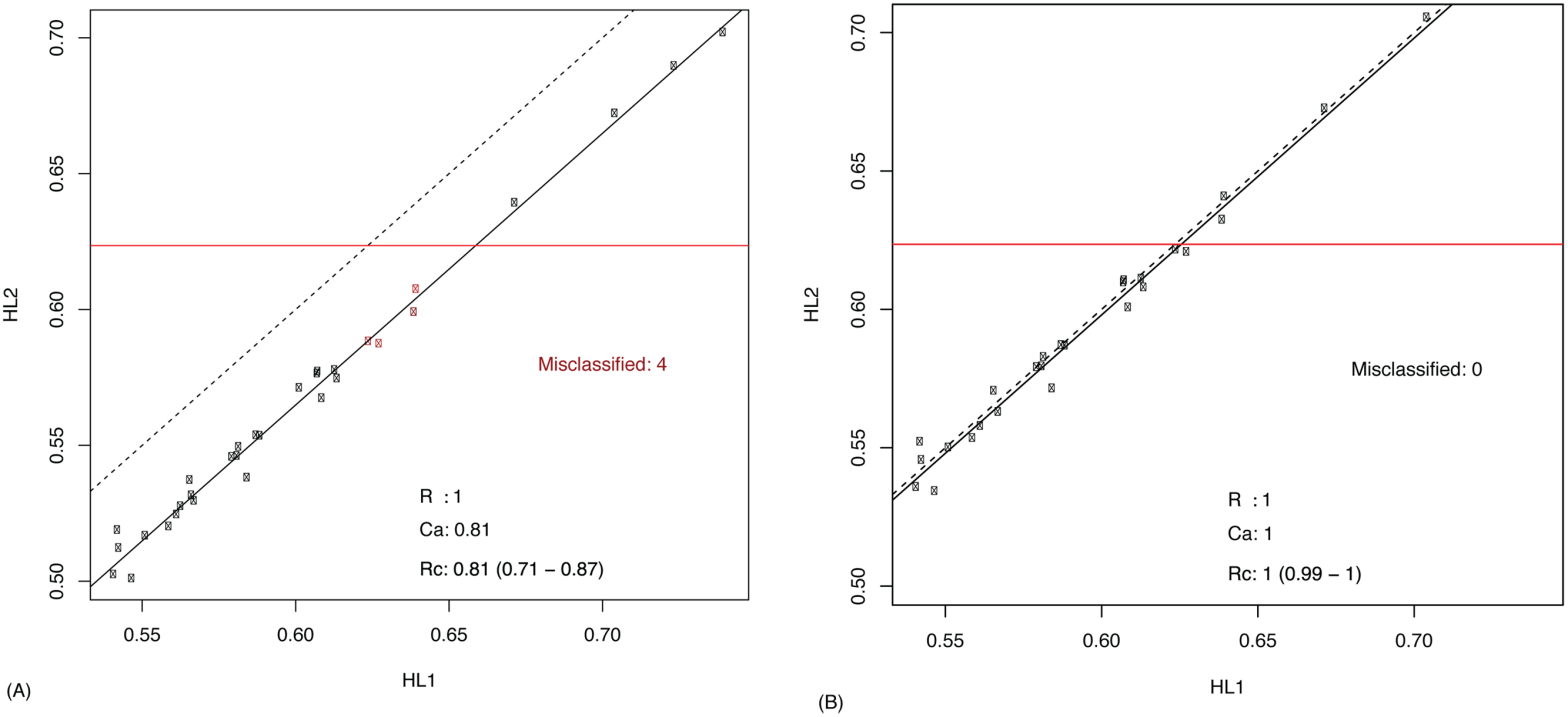
Impact of BE on downstream analysis, illustrated using a HL prognostic model. The x and y axes correspond to risk scores obtained in HL1 and HL2 respectively. The dashed line represents the identity line, and the solid line represents the best linear fit. The horizontal line indicates the threshold used for prediction. The results in (A) correspond to scores not corrected for BE, and in (B) scores are corrected using 3 reference samples that were run in both CodeSets.

BE can have massive implications on downstream analysis. To assess this impact using the NanoString nCounter data, we evaluated a reference-based approach to correct for BE using a previously developed and published HL prognostic model[26] that uses 26 genes to predict recurrence risk score for HL. A predictive score > 0.6235 is used to indicate a higher risk of death. Using this model, we computed risk scores of the same 31 patients represented in two HL CodeSets and compared the concordance of the scores (Fig 7a). While there was an excellent correlation between scores, a lack of accuracy (C_a_ = 0.81) appeared to result in a systematic shift from the identity line. If the threshold model is to be used 4/31 cases would be misclassified as low risk in the second CodeSet when in fact they were classified as high risk in the first CodeSet. This can only be attributed to batch effect associated with CodeSet. We used the reference-based strategy, randomly selecting 3 samples and setting them as a reference (any3_ref), to correct this bias, resulting in no misclassifications (Fig 7b). To ensure that this result was not due to chance, we repeated the process of selecting 3 samples at random and setting them as reference (in both CodeSets) 5000 times. Each time, we corrected for BE and counted the number of misclassification. Over 99% of the times, the resulting C_a_ was near perfect (over 0.99). We observed no misclassification 39% of times, 1 misclassification 18% of the times, and a maximum of 2 misclassifications 44% of the times. It should be noted that in all cases misclassifications were attributed to the same two cases that sat very close to the threshold, and slight perturbations in the data shifted their classification.

## Discussion

We have provided empirical evidence that the use of our reference-based BE correction strategy is equivalent to population-based correction methods with the advantage of being better suited to clinical applications. Though multi-sample BE correction and normalization methods may be appropriate for exploratory retrospective studies, as bulk data would likely be available, special care should be taken to ensure that samples are from homogenous populations. One problematic example within the spectrum of OC could be comparing a North American population OC cohort to a Japanese cohort as the latter are known to have a larger proportion of ovarian clear cell carcinoma histotype and lesser proportion of high-grade serous[38].

While in some cases a biological signal will over-power any of the above noted variability, this is near impossible to predict in advance. Careful experimental design is critical to maximize the utility of any study intending to generate a clinically relevant classifier, whether diagnostic, prognostic or predictive. In such studies, planning for a reference-based strategy is highly desirable. Optimally, a reference would be included on each cartridge—this being the smallest “batch”. However, within-lot variability, examined in several recent studies, were found to be highly stable and to yield negligible effects[23,27]; our data are largely consistent with these findings. This suggests most applications will lend well to periodic reference runs, at least until a locked-down protocol is established for validation[17].

Variability in gene expression data can be partitioned into two sources[39]: biological, caused by differences between different biological conditions, and technical, which can be introduced by virtually every experimental detail. NanoString workflow automation and foregoing the need for enzymatic processing/amplification minimizes technical variability that would otherwise be influenced by the user. For nCounter assays, large lots of reagent are typically ordered upfront and used for all experiments in a given plan. We focused our efforts on evaluating CodeSet-to-CodeSet variability, a topic that has garnered relatively little attention, as well as deriving batch-independent quality assurance metrics and an algorithm for normalization such that assays could be run in small batches, or single patient environments, as one would encounter in clinical practice.

Overall we found lot-to-lot variance on a per-probe basis varied widely and was the single greatest source of identifiable variation. Not all probes varied, however, since we do not have knowledge on batch-manufacture of specific probe sets, it is unknown whether stability is inherent to a gene/probe design or if genes with little difference were from the same manufacturing pool.

In validating our reference-based strategy for batch-independent normalization we encountered some unexpected results. Specifically, the poor performance of the synthetic DNA oligonucleotide references was surprising. This may have been due to: i) A substantial fraction of n-1 and other incomplete oligonucleotides known to occur in longer synthesis reactions even with high coupling efficiency[40]. This may be correctable using HPLC purification, a cost restrictive addition during our experiments. ii) Secondary structure interaction of native RNA that is lacking in DNA. However, given the consistency between signal obtained between replicates of intact mRNA and fragmented mRNA from FFPE sources, we do not suspect significant secondary structure interaction. iii) Unexpected interaction of DNA-oligonucleotides with the colour-barcode molecules. Maintaining consistency in the probe-barcode combinations from batch-to-batch may alleviate this. iv) Other non-optimal hybridization parameters (temperature/salt/detergent) for the probe-DNA duplex compared to probe-RNA duplex of a true sample.

Similarly, in the OC cohort, the cell line-based reference performed poorly. In retrospect, this may have been predictable since many of the genes were not expressed in the cell lines; expression in clinical samples originated from stroma and their absence in cell lines hindered correction.

Finally, we were unable to test specifically whether synthetic RNA-oligonucleotides had superior performance to DNA-oligonucleotides (or other tested references). Synthetic RNA may be an optimal solution for clinical/commercial assays as it could be recreated within very precise parameters. An *in vitro* transcribed RNA strategy appears to be the method employed for NanoString’s FDA approved “ProSigna” assay[41]. However, synthetic RNA pools may be overly expensive to establish and maintain during research and development phases.

## Conclusion

Advance planning is key to any study and identifying the goals in a NanoString-based experiment is no exception. Should a normalization reference sample be required, then careful selection of such a reference should be made: (1) must act identically to a test specimen in the assay, (2) must be plentiful, even in the initial research setting it should last through several CodeSets worth of reagents—stock should be established early, (3) must adequately represent all genes of interest. Cell lines may be suitable in some applications, though not in the above example. If they are used, it is important to avoid replenishing stock by growing more cells as transcript levels may be affected by culture conditions including confluence, nutrient availability, oxygenations, pH, and handling. A new batch of cells is a “new” reference, albeit with similar characteristics to the original. Any new reference must be migrated into the experimental protocol appropriately. Finally, (4) normal tissue, pooled samples or synthetic RNA pools may be considered—it appears that synthetic DNA oligonucleotide pools are not suitable for this purpose. The current *de facto* gold standard, *in vitro* transcribed RNA pools, has been defined by FDA approvals of NanoString’s ProSigna assay. This synthetic RNA approach is unfortunately expensive to establish and maintain during the research and development phase. Large pools of high-quality RNA from real biological samples of interest may be a low cost stand-in, could be shared with others continuing/reproducing results, and can be migrated to replacement pools or a long-term, commercial-product solution at a later stage.

Overall, we found the NanoString gene expression platform to be an easy to use and highly robust technology. Experimental variability is relatively small and can be dealt with using good experimental design and proactive planning, keeping in mind the goals of a given project and the desired reproducibility.

## Supporting Information

S1 File Metrics for Quality Assurance. This file contains additional extensive detail on all methods and parameters related to processing and establishment of metrics for quality assurance.
(DOCX)

S2 File Normalization. This file contains additional extensive detail on the method used for normalization of the data using the reference-based strategy and example data comparing our method to the manufacturer recommendations.
(DOCX)

## References

[1] Jens-Uwe B, Nadia H, Christian J, Ulrike N, Michael U. Evolution of 21-gene Assay Oncotype DX^®^ from an Experimental Assay to an Instrument Assisting in Risk Prediction and Optimisation of Treatment Decision-making in Early Breast. Eur Oncol. 2009;6(1):36–42.

[2] Slodkowska EA, Ross JS. MammaPrint 70-gene signature: another milestone in personalized medical care for breast cancer patients. Expert Rev Mol Diagn. 2009;9(5):417–22. 10.1586/erm.09.32 19580427

[3] Deng MC, Halpern B, Wolters H, Cadeiras M, Hicks A, Rowe T, et al 470: Patient-Specific Longitudinal Patterns of AlloMap Test Scores—Path towards Personalized Medicine? The Journal of Heart and Lung Transplantation. 2009 p. S229.

[4] Vargas J, Lima JAC, Kraus WE, Douglas PS, Rosenberg S. Use of the Corus^®^ CAD Gene Expression Test for Assessment of Obstructive Coronary Artery Disease Likelihood in Symptomatic Non-Diabetic Patients. PLoS Curr. 2013 1;5. 10.1371/currents.eogt.0f04f6081905998fa92b99593478aeab PMC3770834 24043473

[5] Shi L, Tong W, Goodsaid F, Frueh FW, Fang H, Han T, et al QA/QC: challenges and pitfalls facing the microarray community and regulatory agencies. Expert Rev Mol Diagn. Informa HealthcareLondon; 2004 11 9;4(6):761–77. 10.1586/14737159.4.6.761 15525219

[6] Qiaozhen L, Xiaoyang Z, McIntosh T, Davis H, Nemeth JF, Pendley C, et al Development of different analysis platforms with LC-MS for pharmacokinetic studies of protein drugs. Anal Chem. 2009;81(21):8715–23. 10.1021/ac901991x 19788250 PMC2788961

[7] Shi L, Campbell G, Jones WD, Campagne F, Wen Z, Walker SJ, et al The MicroArray Quality Control (MAQC)-II study of common practices for the development and validation of microarray-based predictive models. Nat Biotechnol. 2010 8;28(8):827–38. 10.1038/nbt.1665 20676074 PMC3315840

[8] Leek JT, Scharpf RB, Bravo HC, Simcha D, Langmead B, Johnson WE, et al Tackling the widespread and critical impact of batch effects in high-throughput data. Nat Rev Genet. Nature Publishing Group; 2010 10;11(10):733–9. 10.1038/nrg2825 PMC3880143 20838408

[9] Luo J, Schumacher M, Scherer a, Sanoudou D, Megherbi D, Davison T, et al A comparison of batch effect removal methods for enhancement of prediction performance using MAQC-II microarray gene expression data. Pharmacogenomics J. 2010 8;10(4):278–91. 10.1038/tpj.2010.57 20676067 PMC2920074

[10] Parker HS, Leek JT. The practical effect of batch on genomic prediction. Stat Appl Genet Mol Biol. 2012 1 16;11(3). 10.1515/1544-6115.1766 PMC3760371 22611599

[11] Lazar C, Meganck S, Taminau J, Steenhoff D, Coletta A, Molter C, et al Batch effect removal methods for microarray gene expression data integration: a survey. Brief Bioinform. 2012;bbs037 –. 10.1093/bib/bbs037 22851511

[12] Walker WL, Liao IH, Gilbert DL, Wong B, Pollard KS, McCulloch CE, et al Empirical Bayes accomodation of batch-effects in microarray data using identical replicate reference samples: application to RNA expression profiling of blood from Duchenne muscular dystrophy patients. BMC Genomics. 2008 1;9:494 10.1186/1471-2164-9-494 18937867 PMC2576259

[13] Leek JT, Storey JD. Capturing heterogeneity in gene expression studies by surrogate variable analysis. PLoS Genet. 2007 9;3(9):1724–35. 17907809 10.1371/journal.pgen.0030161 PMC1994707

[14] Johnson WE, Li C, Rabinovic A. Adjusting batch effects in microarray expression data using empirical Bayes methods. Biostatistics. 2007 1;8(1):118–27. 16632515 10.1093/biostatistics/kxj037

[15] Gagnon-Bartsch J a, Speed TP. Using control genes to correct for unwanted variation in microarray data. Biostatistics. 2012 7;13(3):539–52. 10.1093/biostatistics/kxr034 22101192 PMC3577104

[16] Novoradovskaya N, Whitfield ML, Basehore LS, Novoradovsky A, Pesich R, Usary J, et al Universal Reference RNA as a standard for microarray experiments. BMC Genomics. 2004 3 9;5(1):20 15113400 10.1186/1471-2164-5-20 PMC394318

[17] Micheel CM, Nass SJ, Omenn GS, Policy HS. Evolution of Translational Omics Lessons Learned and the Path Forward. 2012. 24872966

[18] Patil P, Bachant-Winne P-O, Haibe-Kains B, Leek JT. Test set bias affects reproducibility of gene signatures. Bioinformatics. 2015;(3):1–6. 10.1093/bioinformatics/btv157 PMC4495301 25788628

[19] Geiss GK, Bumgarner RE, Birditt B, Dahl T, Dowidar N, Dunaway DL, et al Direct multiplexed measurement of gene expression with color-coded probe pairs. Nat Biotechnol. 2008 3;26(3):317–25. 10.1038/nbt1385 18278033

[20] Prokopec SD, Watson JD, Waggott DM, Smith AB, Wu AH, Okey AB, et al Systematic evaluation of medium-throughput mRNA abundance platforms. RNA. 2013 1;19(1):51–62. 10.1261/rna.034710.112 23169800 PMC3527726

[21] Malkov V a, Serikawa K a, Balantac N, Watters J, Geiss G, Mashadi-Hossein A, et al Multiplexed measurements of gene signatures in different analytes using the Nanostring nCounter Assay System. BMC Res Notes. 2009 1;2:80 10.1186/1756-0500-2-80 19426535 PMC2688518

[22] Reis PP, Waldron L, Goswami RS, Xu W, Xuan Y, Perez-Ordonez B, et al mRNA transcript quantification in archival samples using multiplexed, color-coded probes. BMC Biotechnol. BioMed Central Ltd; 2011 1;11(1):46. 10.1186/1472-6750-11-46 PMC3103428 21549012

[23] Veldman-Jones MH, Brant R, Rooney C, Geh C, Emery H, Harbron CG, et al Evaluating Robustness and Sensitivity of the NanoString Technologies nCounter Platform to Enable Multiplexed Gene Expression Analysis of Clinical Samples. Cancer Res. 2015 6 11;0008–5472.CAN—15–0262—. 10.1158/0008-5472.CAN-15-0262 26069246

[24] Kit P. Prosigna ^™^ Breast Cancer Prognostic Gene Signature Assay INTENDED USE / PURPOSE Principles of the nCounter Analysis System. 2013;(Fig 1):1–21.

[25] Northcott P a, Shih DJH, Remke M, Cho Y-J, Kool M, Hawkins C, et al Rapid, reliable, and reproducible molecular sub-grouping of clinical medulloblastoma samples. Acta Neuropathol. 2012 4;123(4):615–26. 10.1007/s00401-011-0899-7 22057785 PMC3306784

[26] Scott DW, Chan FC, Hong F, Rogic S, Tan KL, Meissner B, et al Gene expression-based model using formalin-fixed paraffin-embedded biopsies predicts overall survival in advanced-stage classical Hodgkin lymphoma. J Clin Oncol. 2013 2 20;31(6):692–700. 10.1200/JCO.2012.43.4589 23182984 PMC3574267

[27] Nielsen T, Wallden B, Schaper C, Ferree S, Liu S, Gao D, et al Analytical validation of the PAM50-based Prosigna Breast Cancer Prognostic Gene Signature Assay and nCounter Analysis System using formalin-fixed paraffin-embedded breast tumor specimens. BMC Cancer. BMC Cancer; 2014 1;14(1):177. 24625003 10.1186/1471-2407-14-177 PMC4008304

[28] Anglesio MS, Wiegand KC, Melnyk N, Chow C, Salamanca C, Prentice LM, et al Type-specific cell line models for type-specific ovarian cancer research. PLoS One. 2013;8(9):e72162 10.1371/journal.pone.0072162 24023729 PMC3762837

[29] Altman N. Batch Effects and Noise in Microarray Experiments: Sources and Solutions. Batch Eff noise microarray Exp sources Solut. 2009;34.

[30] TCGA Batch Effects: Overview [Internet]. [cited 2015 Sep 25]. Available from: http://bioinformatics.mdanderson.org/main/TCGABatchEffects:Overview

[31] Köbel M, Kalloger SE, Boyd N, McKinney S, Mehl E, Palmer C, et al Ovarian carcinoma subtypes are different diseases: implications for biomarker studies. PLoS Med. 2008 12 2;5(12):e232 10.1371/journal.pmed.0050232 19053170 PMC2592352

[32] Lin L, Torbeck LD. Coefficient of accuracy and concordance correlation coefficient: new statistics for methods comparison. PDA J Pharm Sci Technol. 1998;52(2):55–9. 9610168

[33] Tothill RW, Tinker A V, George J, Brown R, Fox SB, Lade S, et al Novel molecular subtypes of serous and endometrioid ovarian cancer linked to clinical outcome. Clin Cancer Res. 2008 8 15;14(16):5198–208. 10.1158/1078-0432.CCR-08-0196 18698038

[34] Cancer T, Atlas G. Integrated genomic analyses of ovarian carcinoma. Nature. 2011 6 30;474(7353):609–15. 10.1038/nature10166 21720365 PMC3163504

[35] Technologies N, Ave F. Expression Data Analysis Guide. 2000;

[36] Vandesompele J, De Preter K, Pattyn F, Poppe B, Van Roy N, De Paepe A, et al Accurate normalization of real-time quantitative RT-PCR data by geometric averaging of multiple internal control genes. Genome Biol. 2002;3(7):RESEARCH0034 12184808 10.1186/gb-2002-3-7-research0034 PMC126239

[37] Chen C, Grennan K, Badner J, Zhang D, Gershon E, Jin L, et al Removing batch effects in analysis of expression microarray data: an evaluation of six batch adjustment methods. PLoS One. 2011 1;6(2):e17238 10.1371/journal.pone.0017238 21386892 PMC3046121

[38] Sung P-L, Chang Y-H, Chao K-C, Chuang C-M. Global distribution pattern of histological subtypes of epithelial ovarian cancer: a database analysis and systematic review. Gynecol Oncol. 2014 5;133(2):147–54. 10.1016/j.ygyno.2014.02.016 24556058

[39] Chen JJ, Delongchamp RR, Tsai C-A, Hsueh H, Sistare F, Thompson KL, et al Analysis of variance components in gene expression data. Bioinformatics. 2004;20(9):1436–46. 14962916 10.1093/bioinformatics/bth118

[40] Temsamani J, Kubert M, Agrawal S. Sequence identity of the n-1 product of a synthetic oligonucleotide. Nucleic Acids Res. 1995 6 11;23(11):1841–4. 7596808 10.1093/nar/23.11.1841 PMC306952

[41] NANO46 Genes and Methods to Predict Breast Cancer Outcome. 2013.

