# Supplementary material for: Single-Patient Molecular Testing with NanoString nCounter Data Using a Reference-Based Strategy for Batch Effect Correction
Source: PLoS One. 2016 Apr 20;11(4):e0153844. doi: 10.1371/journal.pone.0153844 (PMC4838303; doi:10.1371/journal.pone.0153844)
Supplement: S1 File — This file contains additional extensive detail on all methods and parameters related to processing and establishment of metrics for quality assurance. (DOCX) [file pone.0153844.s001.docx]

# Supplementary File S1: Metrics for Quality Assurance

Prior to analysis or normalization, the NanoString nCounter data is assessed for technical quality control, in order to gauge the strength of the signal in the data as well as quantify the level of background noise and technical failures. For that, we used the collection of metrics described below.

## Fields Of View (FOV)

The nCounter Digital Analyzer images each lane in discrete units, called fields of view (FOV). The user sets the number of fields of view requested and the Digital Analyzer reports the number of FOVs successfully imaged as FOV Counted. A low percentage of FOV counted could be indicative of a problem with imaging performance. NanoString recommends flagging (or excluding) samples with FOV less than 75%. A given FOV is rejected if no fluorescent barcode can be distinguished, for example due to focus errors, foreign object blocking the lane, or oversaturation of the lane (unable to distinguish one barcode from another).


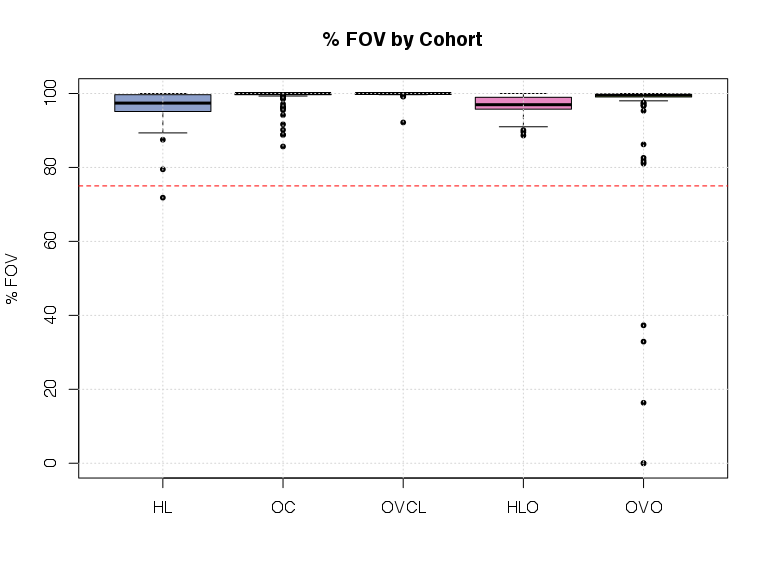


Figure S1: All runs that failed imaging QC based on percent fields of view (FOV) counted across cohorts. HL: Hodgkin lymphoma clinical samples; OC: Ovarian cancer clinical samples; OVCL: ovarian cancer cell lines; HLO: Hodgkin lymphoma DNA oligonucleotides; OVO: Ovarian cancer DNA oligonucleotides.

From Figure S1 and Table S1, failures in the % FOV are noted in one of the duplicated samples in the HL cohort and in five OVO cases. In the OVO cohort the failures are from samples that have functional concentration larger than 222 fM (functional concentration is the total concentration of DNA oligonucleotide for which there was a corresponding NanoString probe in a given experiment/CodeSet). The high concentration of RNA in those samples catastrophically saturated the lanes and resulted in very few fields of view being successfully imaged.

|  | Total | HL | OC | OVCL | HLO | OVO |
| --- | --- | --- | --- | --- | --- | --- |
| **# Runs** | 561 (100%) | 74 (13%) | 258 (46%) | 26 (5%) | 68 (12%) | 135 (24%) |
| **% FOV** |  |  |  |  |  |  |
| mean | 98 ± 0 | 96 ± 1 | 100 ± 0 | 100 ± 0 | 97 ± 0 | 96 ± 1 |
| median | 100 | 97 | 100 | 100 | 97 | 100 |
| IQR | 99 to 100 | 95 to 100 | 100 to 100 | 100 to 100 | 96 to 99 | 99 to 100 |
| range | 0 to 100 | 72 to 100 | 86 to 100 | 92 to 100 | 89 to 100 | 0 to 100 |
| **FOV QC (Failed if %FOV < 75%)** | | | | | | |
| Failed | 6 (1%) | 1 (1%) | 0 (0%) | 0 (0%) | 0 (0%) | 5 (4%) |
| Passed | 555 (99%) | 73 (99%) | 258 (100%) | 26 (100%) | 68 (100%) | 130 (96%) |

Table S1: Summary of imaging QC metrics based on % fields of view (FOV) counted compared across cohorts.

HL: Hodgkin lymphoma clinical samples; OC: Ovarian cancer clinical samples; OVCL: ovarian cancer cell lines; HLO: Hodgkin lymphoma DNA oligonucleotides; OVO: Ovarian cancer DNA oligonucleotides.

## Positive Controls

#### Linearity of Positive Controls

Positive control spike-ins are used to assess the assay linearity. This is achieved by computing $R^{2}$ after fitting a linear model through the raw positive control counts. NanoString recommends flagging assays with $R^{2}<0.95$. When samples are present in too high concentrations, they begin to compete with the high positive controls for lane space, resulting in a loss in linearity.


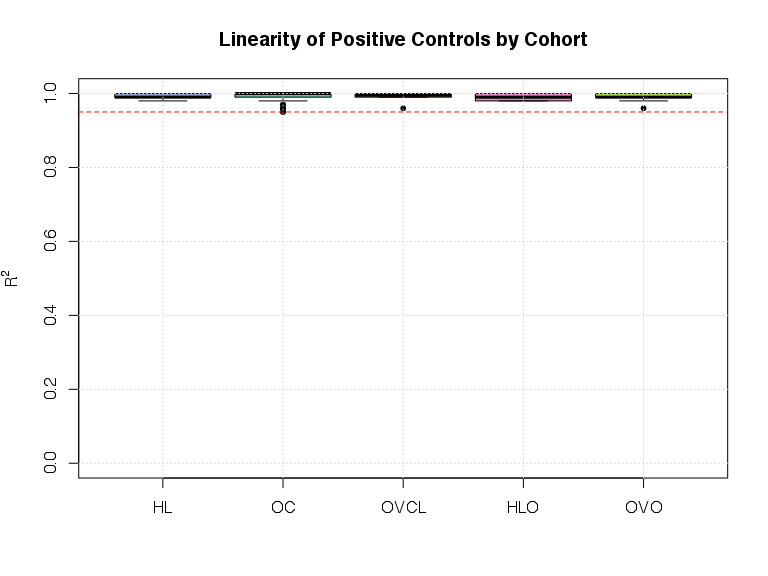


Figure S2: Plot of $\mathbf{R}^{\mathbf{2}}$ of postive control probles from runs across all cohorts. HL: Hodgkin lymphoma clinical samples; OC: Ovarian cancer clinical samples; OVCL: ovarian cancer cell lines; HLO: Hodgkin lymphoma DNA oligonucleotides; OVO: Ovarian cancer DNA oligonucleotides.

Table S2: Distribution of $\mathbf{R}^{\mathbf{2}}$ across the cohorts. All the failures are seen in the OVO dilution experiment.

|  | Total | HL | OC | OVCL | HLO | OVO | |  |
| --- | --- | --- | --- | --- | --- | --- | --- | --- |
| **#Runs** | 561 (100%) | 74 (13.2%) | 258 (46%) | 26 (4.6%) | 68 (12.1%) | | 135 (24.1%) | |
| $\mathbf{R}^{\mathbf{2}}$ |  |  |  |  |  |  | |  |
| mean | 0.99 ± 0.00 | 0.99 ± 0.00 | 0.99 ± 0.00 | 0.99 ± 0.00 | 0.99 ± 0.00 | 0.99 ± 0.00 | |  |
| median | 1 | 0.99 | 1 | 1 | 0.99 | 0.99 | |  |
| IQR | 0.99 to 1 | 0.99 to 1 | 0.99 to 1 | 0.99 to 1 | 0.98 to 1 | 0.99 to 1 | |  |
| range | 0.95 to 1 | 0.98 to 1 | 0.95 to 1 | 0.96 to 1 | 0.98 to 1 | 0.96 to 1 | |  |
| **Linearity QC (Failed if R2 < 0.95%)** | | | | | | | |  |
| Failed | 2 (0.4%) | 0 (0%) | 0 (0%) | 0 (0%) | 0 (0%) | 2 (1.5%) | |  |
| Passed | 559 (99.6%) | 74 (100%) | 258 (100%) | 26 (100%) | 68 (100%) | 133 (98.5%) | |  |

HL: Hodgkin lymphoma clinical samples; OC: Ovarian cancer clinical samples; OVCL: ovarian cancer cell lines; HLO: Hodgkin lymphoma DNA oligonucleotides; OVO: Ovarian cancer DNA oligonucleotides.

From Figure S2 and Table S2, it appears that a linear fit with $R^{2}<0.95$ is uncommon, indicating good linearity of the assays. In the case of the DNA oligonucleotides experiments, the limits of saturation are specifically tested; therefore, it is not surprising to detect over-saturation failures particularly in the samples with very high functional concentration.

#### Detection of Smallest Positive Control

This metric flags samples where the 0.5fM positive control probe is smaller than 2 standard deviations from the mean of the negative controls probes. A failure of this measure indicates that probes expressed at low levels are not distinguishable from background noise. This can be due to high concentration of RNA, resulting in competition for lane space.

Table S3: Failure of detection of smallest PC across cohorts.

|  | Total | HL | OC | OVCL | HLO | OVO |
| --- | --- | --- | --- | --- | --- | --- |
| **# Runs** | 561 (100%) | 74 (13%) | 258 (46%) | 26 (5%) | 68 (12%) | 135 (24%) |
| **Detection of Smallest Positive Control QC** | | | | | | |
| Failed | 2 (0%) | 0 (0%) | 0 (0%) | 0 (0%) | 0 (0%) | 2 (1%) |
| Passed | 559 (100%) | 74 (100%) | 258 (100%) | 26 (100%) | 68 (100%) | 133 (99%) |

HL: Hodgkin lymphoma clinical samples; OC: Ovarian cancer clinical samples; OVCL: ovarian cancer cell lines; HLO: Hodgkin lymphoma DNA oligonucleotides; OVO: Ovarian cancer DNA oligonucleotides.

From Table S3, the two OVO runs with very high concentration failed this QC. It is important to note that these samples had no measured expression level and are clearly the result of very high functional concentration of RNA.

### Signal to Noise Ratio (S/N)

A measure of signal to noise ratio (S/N) would allow us to remove samples that contain little information and likely to add excessive noise to downstream analysis. We assess the signal in the data by considering the average expression level of the housekeeping genes. The level of noise was measured by the limit of detection (LOD).

#### Level of Housekeeping Genes

The average level of expression of the housekeeping genes can serve as a measure of the signal strength, particularly to help assess the amount of useable RNA present in the sample. Generally speaking, housekeeping genes are genes that are not expected to vary between samples. The manufacturer recommends including between 5-8 housekeeping genes in each CodeSet. These should be selected carefully as to not have any association with the biological process under study.


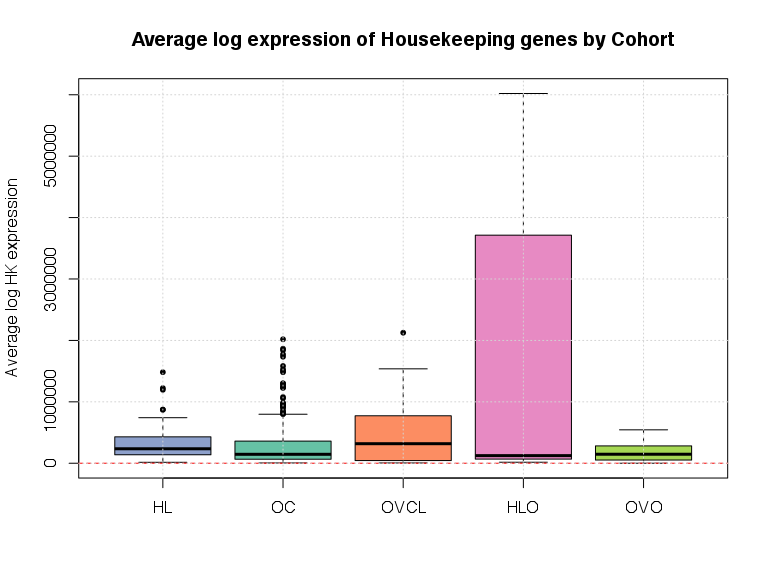


Figure S3: Average log expression of Housekeeping genes by Cohort. HL: Hodgkin lymphoma clinical samples; OC: Ovarian cancer clinical samples; OVCL: ovarian cancer cell lines; HLO: Hodgkin lymphoma DNA oligonucleotides; OVO: Ovarian cancer DNA oligonucleotides.

From Figure S3, we note that the HL DNA oligonucleotides samples exhibit a higher average expression level. This could be attributed to some systematic source of variability present in the data.

#### Limit of Detection (LOD)

The limit of detection (LOD) of negative controls can be used to assess the level of noise in NanoString data. It is computed by taking the arithmetic mean of the raw counts of the eight negative control (NC) probes and adding 2 standard deviations of the NC to the average. This essentially creates an upper bound for the background. The lower the LOD the lower the expected level of background noise in the data.


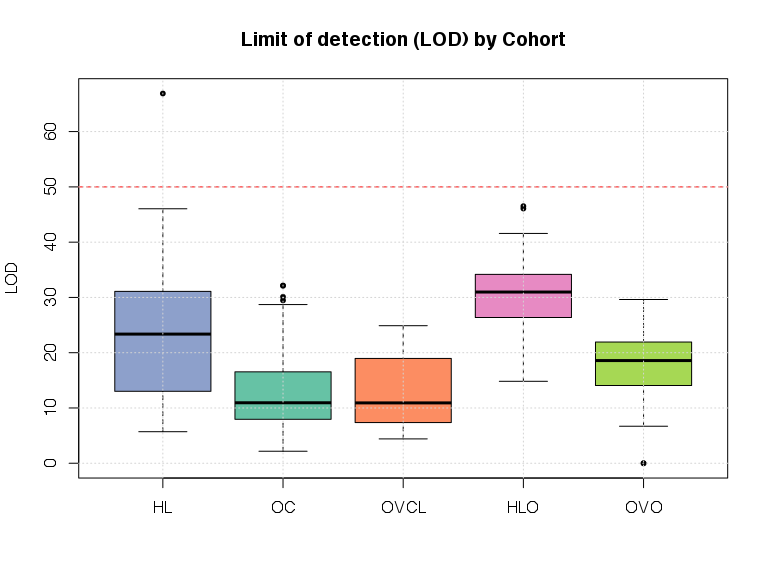


Figure S4: Limit of detection (LOD) by cohort. HL: Hodgkin lymphoma clinical samples; OC: Ovarian cancer clinical samples; OVCL: ovarian cancer cell lines; HLO: Hodgkin lymphoma DNA oligonucleotides; OVO: Ovarian cancer DNA oligonucleotides.

Table S4: Summary statistics of LOD across the different cohorts.

|  | Total | HL | OC | OVCL | HLO | OVO |
| --- | --- | --- | --- | --- | --- | --- |
| **# Runs** | 561 (100%) | 74 (13%) | 258 (46%) | 26 (5%) | 68 (12%) | 135 (24%) |
| **LOD** |  |  |  |  |  |  |
| mean | 17 ± 0 | 24 ± 1 | 12 ± 0 | 12 ± 1 | 31 ± 1 | 18 ± 1 |
| median | 16 | 23 | 11 | 11 | 31 | 19 |
| IQR | 10 to 23 | 13 to 31 | 8 to 17 | 7 to 18 | 26 to 34 | 14 to 22 |
| range | 0 to 67 | 6 to 67 | 2 to 32 | 4 to 25 | 15 to 47 | 0 to 30 |

HL: Hodgkin lymphoma clinical samples; OC: Ovarian cancer clinical samples; OVCL: ovarian cancer cell lines; HLO: Hodgkin lymphoma DNA oligonucleotides; OVO: Ovarian cancer DNA oligonucleotides.

From Figure S4 and Table S4, LOD was generally below 50 for all cases except one sample in the HL cohort.

Given the LOD, we can compute the percent of genes detected, which measures the number of genes with expression above LOD. The number of genes detected generally depends on the underlying biology. However, in this case the genes that are included in each of the CodeSets are expected to have an expression level above background. For this reason, the % gene detected should be relatively large for all samples. When this is not the case, it might be indicative of either the absence of the gene from the sample, or that the RNA is significantly degraded and could not be detected (see **Figure 1**).

Table S5: Descriptive statistics of the % of genes detected above LOD by cohort.

|  | Total | HL | OC | OVCL | HLO | OVO |  |
| --- | --- | --- | --- | --- | --- | --- | --- |
| **# Runs** | 561 (100%) | 74 (13%) | 258 (46%) | 26 (5%) | 68 (12%) | 135 (24%) |  |
| **% Genes Detected** | | | | | | | |
| mean | 93 ± 0 | 85 ± 1 | 93 ± 0 | 69 ± 3 | 100 ± 0 | 97 ± 1 |  |
| median | 97 | 87 | 95 | 74 | 100 | 100 |  |
| IQR | 89 to 100 | 83 to 89 | 90 to 97 | 62 to 79 | 100 to 100 | 100 to 100 |  |
| range | 0 to 100 | 66 to 99 | 60 to 100 | 29 to 90 | 100 to 100 | 0 to 100 |  |

HL: Hodgkin lymphoma clinical samples; OC: Ovarian cancer clinical samples; OVCL: ovarian cancer cell lines; HLO: Hodgkin lymphoma DNA oligonucleotides; OVO: Ovarian cancer DNA oligonucleotides.

The signal to noise ratio can be calculated as a ratio between the geometric mean of housekeeping genes and lower limit of detection: geometric mean/LOD. It can serve as a general measure of how much signal is left in the data after adjusting for the level of noise.

Table S6: Samples that have failed the signal to noise ratio quality measure by cohort.

|  | Total | HLD | OVD | OVCL | HLO | OVO |
| --- | --- | --- | --- | --- | --- | --- |
| **#Runs** | 561 (100%) | 74 (13%) | 258 (46%) | 26 (5%) | 68 (12%) | 135 (24%) |
| **S/N QC (Failed samples are those with S/N <100)** | | | | | | |
| Failed | 12 (2%) | 0(0%) | 0(0%) | 2(8%) | 0(0%) | 10(7%) |
| Passed | 549 (98%) | 74 (100%) | 258(100%) | 24(92%) | 68(100%) | 125(93%) |

HL: Hodgkin lymphoma clinical samples; OC: Ovarian cancer clinical samples; OVCL: ovarian cancer cell lines; HLO: Hodgkin lymphoma DNA oligonucleotides; OVO: Ovarian cancer DNA oligonucleotides.

### Binding Density (BD)

The binding density (BD) is a measure of the number of optical features per square micron. This measure is used for determining whether data collection has been compromised due to image saturation, which generally happens when multiple probes overlap one another. This situation typically arises when RNA is present at a too high concentration.

NanoString recommends flagging samples when the binding density is outside 0.05 - 2.25 range.


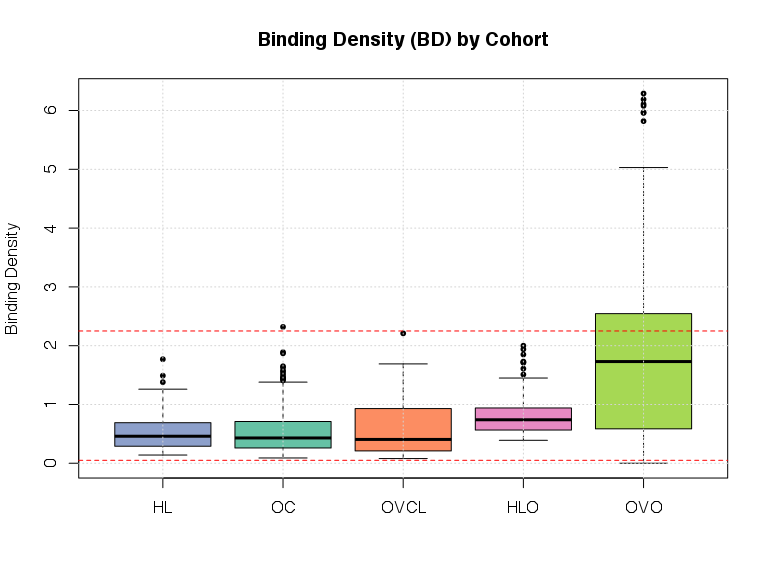


Figure S5: Binding density by cohort. Samples outside of the dashed lines are flagged for having failed the binding density QC measure

Table S7: Binding density summary statistics by cohort.

|  | Total | HL | OC | OVCL | HLO | OVO |
| --- | --- | --- | --- | --- | --- | --- |
| **#Runs** | 561 (100%) | 74 (13.19%) | 258 (45.99%) | 26 (4.63%) | 68 (12.12%) | 135 (24.06%) |
| **BD** |  |  |  |  |  |  |
| mean | 0.92 ± 0.04 | 0.53 ± 0.04 | 0.55 ± 0.02 | 0.62 ± 0.11 | 0.85 ± 0.05 | 1.91 ± 0.13 |
| median | 0.58 | 0.46 | 0.43 | 0.40 | 0.74 | 1.73 |
| IQR | 0.31 to 1.05 | 0.30 to 0.69 | 0.26 to 0.71 | 0.21 to 0.92 | 0.57 to 0.94 | 0.58 to 2.54 |
| range | 0.00 to 6.29 | 0.14 to 1.77 | 0.09 to 2.32 | 0.08 to 2.21 | 0.39 to 2.00 | 0.00 to 6.29 |
| **BD QC** (Failed if outside 0.05 - 2.25 range) | | | | | | |
| Failed | 47 (8.38%) | 0 (0.00%) | 1 (0.39%) | 0 (0.00%) | 0 (0.00%) | 46 (34.07%) |
| Passed | 514 (91.62%) | 74 (100.00%) | 257 (99.61%) | 26 (100.00%) | 68 (100.00%) | 89 (65.93%) |

HL: Hodgkin lymphoma clinical samples; OC: Ovarian cancer clinical samples; OVCL: ovarian cancer cell lines; HLO: Hodgkin lymphoma DNA oligonucleotides; OVO: Ovarian cancer DNA oligonucleotides.

# From Figure S5 and Table S7, most of the failures observed are from the OVO cohort, the ovarian DNA oligonucleotides dilution experiment. As expected, failure was most common in samples with higher functional concentrations. However, this is somewhat inconsistent, as many samples with high functional concentration did not fail the binding density QC metric. We departed from this QC metric in part due to this observation as well as further details discussed below.
