# Supplementary material for: Single-Patient Molecular Testing with NanoString nCounter Data Using a Reference-Based Strategy for Batch Effect Correction
Source: PLoS One. 2016 Apr 20;11(4):e0153844. doi: 10.1371/journal.pone.0153844 (PMC4838303; doi:10.1371/journal.pone.0153844)
Supplement: S2 File — This file contains additional extensive detail on the method used for normalization of the data using the reference-based strategy and example data comparing our method to the manufacturer recommendations. (DOCX) [file pone.0153844.s002.docx]

# Supplementary File S2: Normalization

After filtering out samples that failed quality assurance metrics, the raw nCounter data is log transformed (log base 2) in order to help with distributional assumptions; linearity and consistency with PCR based methods for calculating fold change.

The manufacturer's suggested normalization steps correct technical variations between samples and adjust the gene expression relative to the RNA content of housekeeping genes. The procedures suggested by NanoString depend on a specific order. A first step is to perform lane-to-lane normalization using positive control probes. This is followed by sample content normalization using housekeeping genes, and finally background normalization using Negative controls (detailed in the “NanoString nCounter Expression Data Analysis Guide”, http://www.nanostring.com).

*Positive control normalization* This step accounts for sample-to-sample, or lane-to-lane variability and is done by summarizing the positive control counts using a summary measure such as mean, median, sum or geometric mean. This is used to obtain a scaling factor that is used to adjust samples relative to one another

*Background normalization* Background effect in NanoString data is present and can be attributed to cross hybridization and imaging noise. Background counts are assessed using negative control genes.

*Housekeeping genes normalization* This normalization step is used to adjust counts of experimental probes, relative to specific control probes (housekeeping genes) where it is assumed the expression of housekeeping genes remains constant across all relevant test samples. This normalization serves to eliminate variability between sample-to-sample RNA inputs.

From our analysis of NanoString data, we found that many of these steps are not necessary and moreover, they are not applicable directly in single-patient samples.

#### Positive control normalization is redundant to Housekeeping genes normalization

Many of the suggested normalization steps are redundant and could become problematic in the analysis of a single sample. Housekeeping gene normalization alone is equivalent to positive control normalization with housekeeping gene normalization and does not change the end result. In the figure below, we select a gene at random from the ovarian cancer data and we compare the results of two normalization methods: one using housekeeping genes only (HK) and another using both housekeeping genes and positive control normalization (PC). We repeat this procedure in two CodeSets. We can see within each CodeSet, the two normalization procedures produce near identical results. Hence, we recommend against positive control normalization to avoid processing the data unnecessarily.


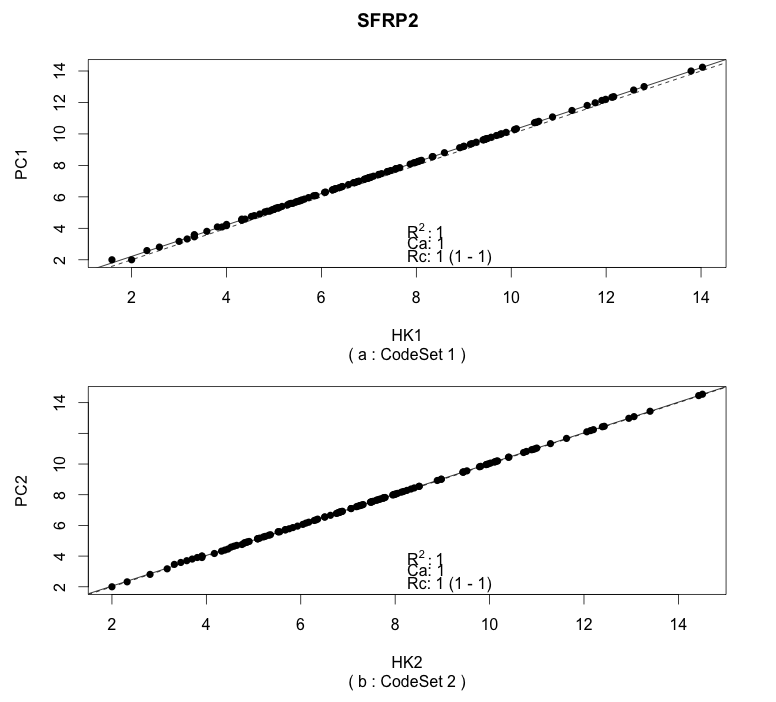


Figure S6 Ovarian Cancer data comparisons of two normalization procedures of the data of the gene SFRP2. The method that performs only housekeeping normalization is denoted by HK1 and HK2 in CodeSet 1 and 2 respectively. On the other hand, the method that normalizes both to positive controls as well as housekeeping genes at the same time, is denoted by PC1 and PC2 when ran on CodeSet 1(a) and CodeSet 2(b) respectively

####

#### Background correction is not necessary

The aim of background correction is to make zero a baseline for “no expression”. In the presence of batch effect, background normalization, in addition to the increased variance that is introduced, becomes particularly difficult because the background threshold is different from batch to batch. In Figure S7, we select a gene at random from the ovarian cancer data and we compare the results of two normalization methods; one method uses housekeeping genes only (HK) and another uses both housekeeping genes and negative control normalization to mean plus 2 standard deviations (NC). We repeat this procedure in two CodeSets. We can see within each CodeSet, the two normalization procedures produce similar results, however, it is apparent that an artifact of background correction is clearly present. In order to avoid including this artifact, we recommend avoiding background correction in general.


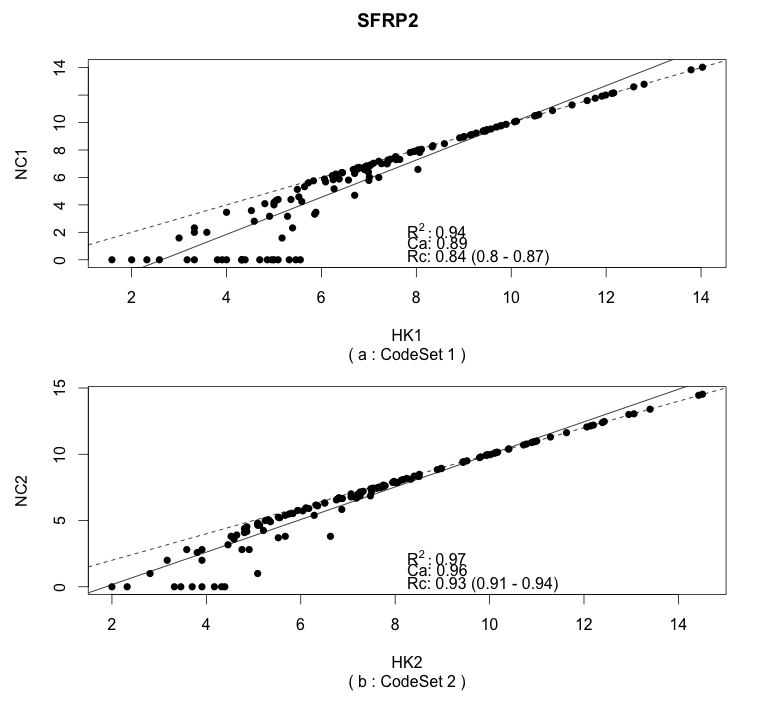


Figure S7 Ovarian Cancer data comparisons of two normalization procedures of the data of the gene SFRP2. The method that performs only housekeeping normalization is denoted by HK1 and HK2 in CodeSet 1 and 2 respectively. On the other hand, the method that normalizes housekeeping genes as well as performs background correction, is denoted by NC1 and NC2 when ran on CodeSet 1(a) and CodeSet 2(b) respectively.

#### Choice of Housekeeping Genes

The choice of housekeeping genes is very important. Optimal housekeeping genes should be correlated with one another and independent of the biological signal of interest. Furthermore, it is desirable that they have a moderately high expression level (beyond the point of minimal detection) and a smaller variance. A good housekeeping gene would have relatively high mean expression and low standard deviation across samples. They should be at the lower right hand corner of the mean versus standard deviation plot. This plot can be used to identify other possible housekeeping genes.


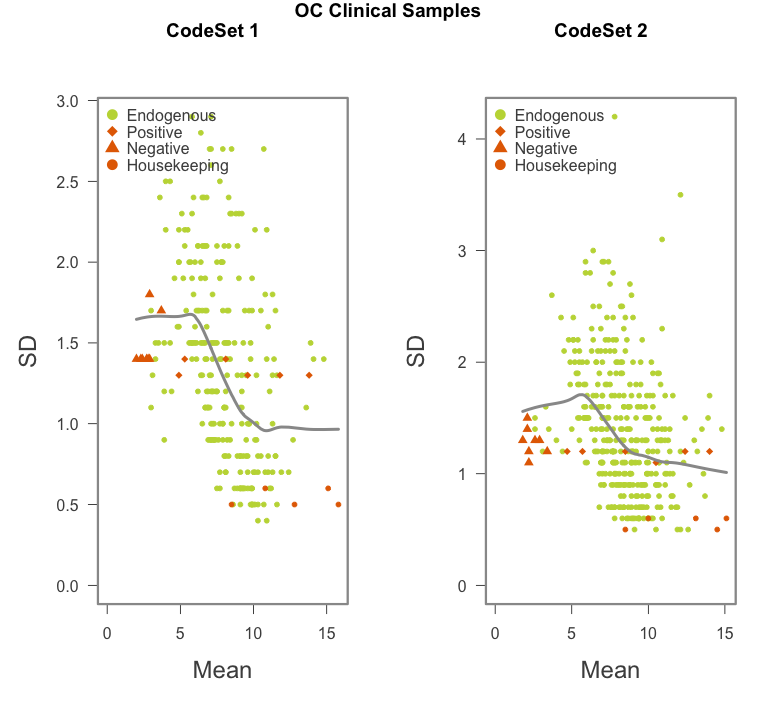


Figure S8 In all datasets we can see that where the negative control genes are is the lower limit of detection. This is different for different CodeSets. The housekeeping genes have constant standard deviation, which is also different in different CodeSets. Finally the standard deviation of housekeeping genes is low relative to other genes which indicates that the genes do not fluctuate greatly between conditions

#### Correction using Housekeeping Genes without scaling by overall mean.

In a single-sample correction method, it is important to be able to adjust the expression level for each sample, using only the information obtained from that sample. The method suggested by NanoString scales the ratios by a factor equivalent to the mean of all the genes run in a CodeSet. This is equivalent to shifting by all the values by the mean on the log scale. This is done in order to put everything back near the original scale to allow ease of interpretation. However, this step is unnecessary and may introduce bias through dependence on samples run simultaneously within a CodeSet. Furthermore, it alters how the normalized results are interpreted. Without this scaling step they are fold changes over or below housekeeping genes. Once they are shifted by the mean, this interpretation is lost.


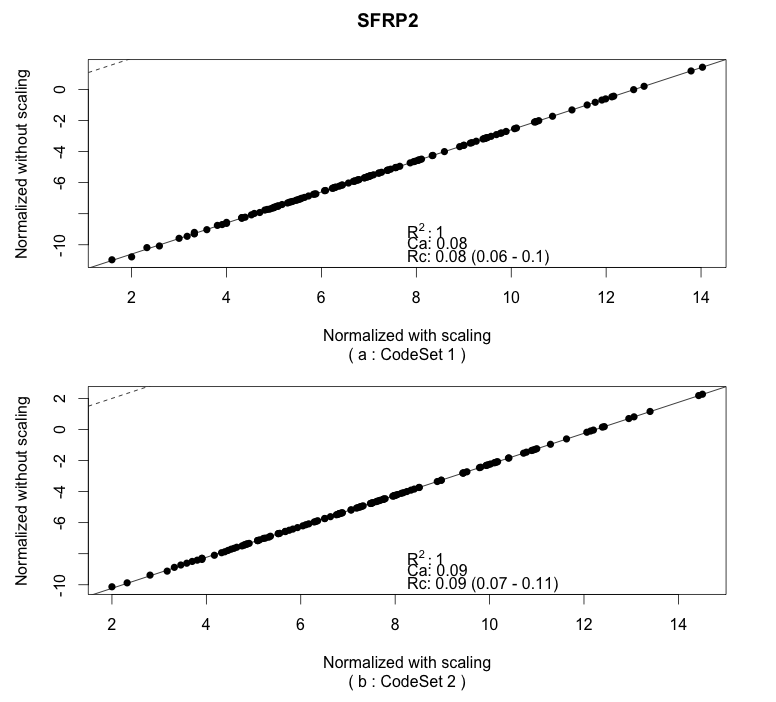


Figure S9 Ovarian Cancer data comparisons of two normalization procedures of the data of the gene SFRP2. On the x-axis is the method that scales the data post normalization and on the y-axis is the method that leaves the expression values as change relative to housekeeping genes. This was done in CodeSet 1(a) and CodeSet 2(b) respectively. The dashed line indicates the identity line.
